# Targeting NME3 to Restore Mitochondrial Fission‐Fusion Balance Defines a Novel Disease‐Modifying Strategy for Parkinson's Disease

**DOI:** 10.1002/cns.70822

**Published:** 2026-03-09

**Authors:** Chen Qiao, Xiang‐Qi Hu, Shen‐Han Xu, Meng‐Fan Yao, Jun‐Peng Liu, Lei Cao

**Affiliations:** ^1^ Department of Clinical Pharmacy, Affiliated Hospital of Jiangsu University Jiangsu University Zhenjiang Jiangsu China; ^2^ College of Pharmacy Jiangsu University Zhenjiang Jiangsu China; ^3^ Jiangsu Key Laboratory of Neurodegeneration, Department of Pharmacology Nanjing Medical University Nanjing China

**Keywords:** disease‐modifying intervention, mitochondrial dynamics, neuroprotection, NME3, Parkinson's disease

## Abstract

**Aims:**

Parkinson's disease (PD) lacks effective disease‐modifying therapies, despite mitochondrial dysfunction being a key pathogenic factor. This study aimed to identify novel regulators of mitochondrial dynamics and explore their therapeutic relevance.

**Methods:**

Transcriptomic analysis was conducted on the substantia nigra (SN) of 1‐methyl‐4‐phenyl‐1,2,3,6‐tetrahydropyridine (MPTP)‐induced PD mice. SN‐specific lentiviral knockdown or overexpression of nucleoside diphosphate kinase 3 (NME3) was performed in mice. Motor behavior, dopaminergic neuron survival, mitochondrial ultrastructure, and reactive oxygen species (ROS) levels were assessed. Mitochondrial fission was pharmacologically inhibited using the Drp1 inhibitor Mdivi‐1.

**Results:**

RNA sequencing revealed a marked reduction of *Nme3* in the SN of MPTP‐treated mice. *Nme3* knockdown in healthy mice induced PD‐like motor deficits and dopaminergic neurodegeneration, mimicking the MPTP model. Mechanistically, NME3 deficiency disrupted mitochondrial fission‐fusion balance, causing abnormal mitochondrial morphology, excessive ROS production, and neuronal injury. Mdivi‐1 treatment significantly alleviated mitochondrial dysfunction and neurotoxicity. Conversely, SN‐specific *Nme3* overexpression in MPTP‐treated mice improved motor performance and preserved dopaminergic neurons by suppressing pathological mitochondrial fission.

**Conclusion:**

NME3 is a previously unrecognized regulator of mitochondrial dynamics and a critical contributor to PD pathogenesis. Restoring mitochondrial fission‐fusion balance through genetic or pharmacological approaches provides neuroprotection, highlighting NME3 as a promising target for disease‐modifying PD therapies.

## Graphical abstract

NME3 deficiency disrupts mitochondrial fission‐fusion balance, causing neuronal damage via ROS. Pharmacological Drp1 inhibition (Mdivi‐1) or substantia nigra‐specific NME3 overexpression restores mitochondrial equilibrium, prevents dopaminergic neuron loss, and alleviates motor deficits in PD models. This identifies NME3‐targeted strategies as novel disease‐modifying therapies for Parkinson's disease.

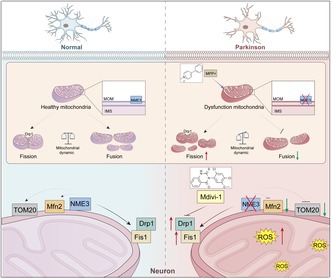

## 1 Introduction

PD is the second most common neurodegenerative disorder, with a prevalence of approximately 0.2% in the global population [1]. The primary pathological features of PD include the selective loss of dopaminergic (DA) neurons in the substantia nigra pars compacta (SNc) and the accumulation of Lewy bodies (LBs) [2, 3]. Current clinical therapies for PD mainly alleviate motor symptoms but fail to slow disease progression [4, 5]. The lack of a cure for PD is largely attributed to an insufficiently precise understanding of its pathogenesis [6, 7]. Therefore, elucidating the pathological mechanisms of PD is urgently needed to provide novel therapeutic targets for drug development.

Neurons play a central role in the pathological progression of PD, with their functional abnormalities and progressive degeneration promoting the onset and development of the disease [8]. Recent studies have revealed molecular mechanisms and regulatory targets involved in neuronal degeneration during PD progression [9, 10]. The degeneration of DA neurons is regulated by multiple mechanisms, including mitochondrial dysfunction, abnormal accumulation of reactive oxygen species (ROS), and neuroinflammatory responses [11, 12]. Research has demonstrated that mitochondrial impairment in DA neurons leads to insufficient energy supply and excessive ROS production, which further damages neurons and ultimately contributes to PD [13]. Genetic factors such as mutations in LRRK2, PINK1, and Parkin exacerbate neuronal damage by disrupting mitochondrial quality control, thereby triggering PD [14]. Therefore, maintaining normal neuronal physiological function is crucial for modulating the pathological progression of PD.

Mitochondria not only provide energy for neurons but also participate in regulating redox homeostasis, iron metabolism, calcium signaling, and apoptosis [15, 16]. Intracellular mitochondria continuously undergo fusion, fission, and degradation, maintaining a dynamic equilibrium under normal physiological conditions [17]. During mitochondrial fusion, Mitofusin 1 (Mfn1) and Mfn2 interact to form both homo‐oligomeric and hetero‐oligomeric complexes, thereby tethering the outer membranes of adjacent mitochondria [18, 19]. Dynamin‐related protein 1 (Drp1) serves as the primary regulator driving mitochondrial fission; its translocation to the mitochondrial outer membrane and subsequent assembly into oligomers with Mitochondrial fission factor (Mff) and Fission 1 (Fis1) induces mitochondrial fission [19, 20]. Impaired mitochondrial dynamics can mediate or amplify mitochondrial and neuronal dysfunction [21]. In most PD pathological models, inhibition of mitochondrial fission has been shown to alleviate mitochondrial dysfunction, indicating the crucial role of mitochondrial dynamics in neurotoxicity induced by PD‐associated mutant proteins [22]. When mitochondrial dynamic homeostasis is disrupted, dysfunctional mitochondria and those generating high levels of reactive oxygen species excessively accumulate, releasing substantial neurotoxic substances that impair the normal physiological functions of DA neurons or even induce their death, ultimately leading to PD [4].

Nucleoside diphosphate kinase 3 (NME3), a member of the Nucleoside diphosphate kinases (NDPK) family, is localized to the outer membrane of mitochondria and has been implicated in the pathological progression of neurodegenerative diseases, particularly through its role in regulating mitochondrial dynamics [23]. Previous studies have shown that NME3 binding to the mitochondrial outer membrane promotes mitochondrial fusion, thus helping maintain normal mitochondrial morphology and function. Specifically, NME3 interacts with phosphatidic acid at the mitochondrial tips via its N‐terminal region, facilitating mitochondrial tethering and stimulating Mfn1/2‐mediated fusion processes [23, 24]. Although mitochondrial dynamics imbalance is recognized as a key mechanism underlying neurodegeneration in PD, current therapeutic strategies targeting mitochondrial damage have not led to satisfactory clinical outcomes. This highlights the urgent need for novel mitochondria‐targeted therapeutic approaches in PD treatment. Despite the known role of NME3 in mitochondrial fusion, no direct evidence has yet linked NME3 to the regulation of PD pathological progression. This gap in knowledge underscores the innovative nature of our study, which aims to explore NME3's involvement in PD and its potential as a therapeutic target for restoring mitochondrial dynamics in DA neurons.

In this study, through transcriptome sequencing combined with PD pathological models, our results demonstrated that NME3 deficiency induced PD‐like motor dysfunction and DA neuron degeneration in mice. Additionally, NME3 deficiency resulted in abnormal mitochondrial morphology and a disrupted mitochondrial fission‐fusion balance. In vitro studies further confirmed that NME3 deficiency caused mitochondrial dynamics imbalance, excessive ROS production, and exacerbated DA neuronal damage. Notably, treatment with the mitochondrial fission inhibitor Mdivi‐1, or overexpression of NME3, successfully reversed the mitochondrial abnormalities induced by NME3 deficiency. Furthermore, midbrain‐specific NME3 overexpression in PD model mice effectively alleviated motor dysfunction and prevented DA neuron loss. This overexpression also restored mitochondrial dynamics balance by suppressing excessive mitochondrial fission and promoting mitochondrial fusion. Taken together, our study offers novel insights into the molecular pathogenesis of PD and highlights NME3 as a potential therapeutic target. These findings lay the groundwork for developing mitochondria‐targeted, disease‐modifying therapies for PD.

## 2 Materials and Methods

### 2.1 Experimental Animals

All experimental animals were approved by the Institutional Animal Care and Use Committee of Nanjing Medical University (IACUC No. 2008067). Male C57BL/6J mice (3–4 months old, specific pathogen‐free grade) weighing 20–27 g were used in this study. To avoid the interference of biological sex, such as estrogen or estrogen receptors, in the development and phenotypic expression of PD, female mice were excluded from this study. The mice were housed under controlled conditions (24°C ± 2°C) with ad libitum access to food and water, and maintained on a 12‐h light/dark cycle.

### 2.2 Mouse Model Preparation

#### 2.2.1 Subacute PD Mouse Model

Male mice were intraperitoneally injected with 1‐methyl‐4‐phenyl‐1,2,3,6‐tetrahydropyridine (MPTP) (Sigma‐Aldrich, Cat# M0896) at a dose of 20 mg/kg once daily for 5 consecutive days. Tissues were collected 12 days after the last injection. Control mice received equivalent volumes of saline following the same injection schedule.

#### 2.2.2 Stereotaxic Intracerebral Injection

*NME3‐knockdown mouse model*: Two weeks prior to model induction, lentivirus LV‐siNME3 was stereotactically injected into the SNc region of the midbrain, while control groups received equivalent injections of negative control virus (LV‐NC).

*NME3‐overexpressing mouse model*: Two weeks before model induction, lentivirus LV‐NME3‐OE was stereotactically injected into the SNc region of the mouse midbrain, while control groups received equivalent injections of negative control virus (LV‐NC).

*Subacute MPTP‐induced PD mouse model with NME3 overexpression*: Specifically, 2 weeks prior to MPTP administration, lentivirus expressing NME3 (LV‐NME3‐OE) was stereotactically injected into the substantia nigra pars compacta (SNc) region of the midbrain. Control mice received equivalent injections of the negative control lentivirus (LV‐NC).

The SNc coordinates were determined according to *The Mouse Brain in Stereotaxic Coordinates (4th edition)*. The SNc is located in the ventral midbrain, dorsal to the red nucleus, ventral to the cerebral peduncle, and partially overlapping with the ventral tegmental area caudally. Using bregma as the reference point, the stereotaxic coordinates were: anterior–posterior (AP) −2.8 to −3.8 mm, medial‐lateral (ML) ±1.0 to 1.5 mm, and dorsal‐ventral (DV) −4.2 to −4.8 mm (measured vertically from the brain surface) (Supplementary Figure 1). All lentiviral constructs were purchased from OBiO Technology (Shanghai) Corp., with the following sequences (5′‐3′):

LV‐siNME3: 5´‐GAGCTACTGCGGGAGCATTAT‐3′;

LV‐NC (NME3 knockdown): 5´‐CCTAAGGTTAAGTCGCCCTCG‐3′;

LV‐NME3‐OE: 5´‐CGCAAATGGGCGGTAGGCGTG‐3′;

LV‐NC (NME3 overexpression): 5′‐AGAGACAGCAACCAGGAT‐3′.

The key experiment showed that there was no significant difference between the completely untreated group (Mock) and the LV‐NC group (Figure S7). Therefore, we used the corresponding LV‐NC as the control group for the subsequent experiments in mice.

### 2.3 Behavioral Tests

Behavioral assessments were conducted using a double‐blind protocol 5 days after the final MPTP injection.

#### 2.3.1 Open Field

The open field test was used to evaluate locomotor activity and exploratory behavior in mice. After 30 min of environmental habituation in the behavioral observation chamber, each mouse was placed in the central zone of the open field. The video tracking system was activated to record the animal's movement trajectories for 5 min.

#### 2.3.2 Rotarod Test

Motor coordination was quantitatively assessed using the rotarod test. During the 2‐day acclimation period, mice were trained at a constant speed of 10 rpm for 5 min. For the formal test, mice were placed on the rotating rod, and the apparatus was activated after stabilization. The rod accelerated linearly from 2 to 20 rpm over 5 min, and the latency to fall was recorded.

#### 2.3.3 Pole Test

The pole test was employed to assess fundamental motor function in mice. The apparatus consisted of a 1‐cm‐diameter wooden pole (50 cm height) wrapped with gauze to prevent slipping, topped with a 25‐mm‐diameter cork ball. During the training phase, mice underwent two consecutive days of daily 1‐min practice sessions. For formal testing, each mouse was placed head‐up at the pole's apex while simultaneously activating the timer, with the following temporal parameters recorded: (1) time from movement initiation to complete body axis reversal, and (2) total descent duration to the pole base.

### 2.4 Nissl Staining

The cryopreserved brain tissue samples were thawed and sectioned according to target brain regions identified using a stereotaxic atlas. Tissue sections were sequentially immersed in xylene, absolute ethanol, and graded ethanol solutions (95%, 80%, and 70%) for 1 min each, followed by a 1‐min rinse in distilled water. Nuclei were then stained with 0.1% cresyl violet solution for 1.5–3 min, immediately followed by a rapid 30‐s distilled water rinse to remove excess dye. Differentiation was performed using absolute ethanol for 30 s to optimize staining quality, after which the sections were dehydrated through graded ethanol solutions. Following 30 min of xylene clearing to enhance tissue transparency, the samples were air‐dried at room temperature and mounted with neutral balsam for microscopic observation and image acquisition using optical microscopy.

### 2.5 Immunohistochemistry and Immunofluorescence Staining

Mouse brain tissues were dehydrated in 20% and 30% sucrose dissolved in PBS for 3 days, followed by fixation in 4% paraformaldehyde. The samples were embedded in OCT compound and sectioned (30 μm) using a Leica cryostat. The sections were stored in PBS containing 50% glycerol at −20°C until use. For immunohistochemistry, sections were washed three times in 0.01 M PBS and incubated in 3% H_2_O_2_ for 30 min to quench endogenous peroxidase activity. After PBS washes, the sections were blocked with 5% bovine serum albumin (prepared in 0.01 M PBS containing 0.3% Triton X‐100) for 1 h at room temperature, followed by incubation with primary antibodies overnight at 4°C. Subsequently, appropriate secondary antibodies were applied for 1 h at room temperature. Immunoreactivity was visualized using DAB substrate. For immunofluorescence, nuclei were counterstained with DAPI. Samples for immunofluorescence and immunohistochemical assays were sections derived from the midbrain of mice. We made continuous 20 μm‐thick sections of the midbrain, taking 1 section every 6 intervals for stereological counting and statistics.

The following primary antibodies were used: mouse anti‐TH antibody (1:160, Invitrogen, Cat# MA1‐24654), rabbit anti‐NME3 antibody (1:1000, Proteintech, Cat# 15136‐1‐AP), mouse anti‐GFAP antibody (1:800, Sigma, Cat# MAB360), mouse anti‐IBA1 antibody (1:1000, Invitrogen, Cat# GT10312), rabbit anti‐Drp1 antibody (1:5000, Proteintech, Cat# 12957‐1‐AP), rabbit anti‐Mfn2 antibody (1:5000, Proteintech, Cat# 12186‐1‐AP), mouse anti‐Tuj1 antibody (1:50, Santa Cruz, Cat# sc‐80,005).

### 2.6 Transmission Electron Microscopy (TEM) of Midbrain Tissue

Following anesthesia, experimental mice underwent cardiac perfusion with pre‐chilled 2% paraformaldehyde‐3% glutaraldehyde fixative (prepared in 0.1 M PBS, pH 7.4). The SNc region was identified according to the Paxinos and Watson mouse brain stereotaxic atlas, and 1 mm^3^ tissue blocks were rapidly dissected and transferred to fresh fixative for 2 h at 4°C. The tissue samples were subsequently dehydrated through an ethanol gradient series followed by absolute ethanol and acetone treatments. Specimens were embedded in epoxy resin and ultrathin sections were prepared using an ultramicrotome. Finally, sections were stained with 2% lead citrate solution for electron microscopic observation (Shanghai Xibailai Biotechnology Co. Ltd.). For each midbrain tissue sample, 8 electron micrographs of different regions were captured randomly, and all micrographs were taken at a fixed magnification and fixed image size to ensure consistency of observation standards among different samples. The captured images were stored in a dedicated computer for subsequent quantitative statistical analysis of mitochondrial morphology (such as mitochondrial morphology, area, and the number of damaged mitochondria). All image capture and statistical processes were performed by two independent researchers who were blinded to the group information to eliminate subjective bias.

### 2.7 SH‐SY5Y Cell Culture, Transfection, and Differentiation

SH‐SY5Y cells (Shanghai Aibimeng Biotechnology Co. Ltd.) were cultured in high‐glucose DMEM (Gibco, Cat# C11995500BT) supplemented with 15% FBS (Gibco, Cat# 04‐001‐1ACS), 100 U/mL penicillin, and 100 U/mL streptomycin (Gibco, Cat# 15140‐122) at 37°C in a 5% CO_2_ humidified incubator. Cells in logarithmic growth phase were counted and seeded in culture plates at approximately 40% confluence prior to transfection with either knockdown (LV‐siNME3) or overexpression (LV‐NME3‐OE) lentiviruses. After 12 h of viral transduction, the medium was replaced with fresh complete medium, followed by continued culture for 48 h before subsequent experiments. Lentiviral constructs (OBiO Technology, Shanghai) included (5′‐3′):

LV‐NME3‐siRNA (Y22377): 5´‐GTTGGCAAGAATGTAATTCAT‐3′;

LV‐NME3‐siRNA (Y22378): 5´‐GAGCTACTGCGGGAGCATTAT‐3′;

LV‐NME3‐siRNA (Y22379): 5´‐CGGACATTGGCTATATTGAGTA‐3′;

LV‐NC (NME3 knockdown): 5´‐CCTAAGGTTAAGTCGCCCTCG‐3′;

LV‐NME3‐OE: 5′‐CGCAAATGGGCGGTAGGCGTG‐3′;

LV‐NC (NME3 overexpression): 5´‐AGAGACAGCAACCAGGAT‐3′.

The key experiment showed that there was no significant difference between the completely untreated group (Mock) and the LV‐NC group (Figure S7). Therefore, we used the corresponding LV‐NC as the control group for the subsequent transfection experiments.

SH‐SY5Y cells were cultured and differentiated following established protocols [25]. Briefly, cells were maintained in basic growth medium consisting of DMEM/F12 supplemented with 10% FBS and 1× penicillin/streptomycin, and seeded onto 6‐well plates pre‐coated with poly‐L‐ornithine and fibronectin at a density of 1.5 × 10^5 cells/well. After 24 h, the medium was replaced with Stage I differentiation medium containing DMEM/F12 with 2.5% FBS, 10 μM retinoic acid, and 1× penicillin/streptomycin, and cells were cultured for 5 days. Subsequently, Stage II differentiation medium, consisting of DMEM/F12 with 1% N2 supplement, 50 ng/mL BDNF, 20 mM KCl, and 1× penicillin/streptomycin, was applied for an additional 5 days. Following successful differentiation into dopamine‐like neurons, cells were subjected to transfection experiments.

This study used undifferentiated SH‐SY5Y cells for mechanistic analyses as previously, while differentiated SH‐SY5Y cells were used to directly evaluate neuronal phenotypes relevant to dopaminergic pathology.

### 2.8 CCK‐8 Cell Viability Assay

Cells in logarit'hmic growth phase were counted and seeded in 96‐well plates at 100 μL/well. Cells were cultured in a 37°C, 5% CO₂ incubator for 24 h until reaching approximately 80% confluence, followed by drug intervention. Following treatment, culture medium was replaced with 100 μL serum‐free medium containing 10 μL CCK‐8 reagent (Beyotime, Cat# S0039), followed by 1 h incubation at 37°C protected from light. Absorbance at 450 nm was measured using a microplate reader for quantitative analysis.

### 2.9 Mitochondrial ROS Detection by Flow Cytometry

Cells were incubated with 200 μL DCFH‐DA working solution (Beyotime, Cat# S0033S; 1:1000 in serum‐free medium) for 20 min at 37°C in the dark. After washing three times with serum‐free medium, cells were resuspended in PBS for flow cytometric analysis.

### 2.10 Measurement of ATP and Lactate Levels

SH‐SY5Y cells were seeded at appropriate density and treated at 70%–80% confluence. Four biological replicates were included per group.

Intracellular ATP levels were measured using an ATP Assay Kit (Beyotime, Cat# S0026) based on the luciferin‐luciferase reaction. Cells were lysed in pre‐cooled ATP lysis buffer, centrifuged at 12,000 rpm for 5 min at 4°C, and supernatants were incubated with luciferin‐luciferase working solution. Luminescence was recorded, and ATP concentrations were calculated from a standard curve.

Extracellular lactate levels were quantified using a Lactic Acid Assay Kit (Nanjing Jiancheng, Cat#A019‐2‐1). Supernatants were collected by centrifugation (8000 rpm, 3 min, 4°C) and reacted with reagents according to the manufacturer's instructions. Absorbance was measured at 530 nm, and lactate concentrations were determined using a standard curve.

### 2.11 Mitochondrial Membrane Potential Assay

JC‐1 working solution was prepared by diluting JC‐1 (Beyotime, Cat# C2003S‐1) 1:200 in JC‐1 buffer (Beyotime, Cat# C2003S‐2). Cells were digested, resuspended, and seeded in culture dishes. After reaching optimal confluency, drug treatments were administered. Following 48 h of treatment, cells were incubated with JC‐1 working solution for 15 min at 37°C, then washed twice with JC‐1 buffer. Coverslips containing cells were mounted onto glass slides with DAPI‐containing antifade mounting medium and incubated for 10 min in the dark prior to image acquisition using fluorescence microscopy.

Differentiated SH‐SY5Y cells were plated on poly‐D‐lysine‐coated coverslips and subjected to the indicated treatments. Following treatment, cells were incubated with TMRM (Thermo Fisher, Cat# I34361) at a final concentration of 200 nM in culture medium for 30 min at 37°C in the dark. After incubation, cells were gently washed twice with PBS to remove excess dye. Fluorescence images were captured immediately using a confocal laser scanning microscope with identical imaging settings for all conditions. Mitochondrial membrane potential was quantified by measuring the mean fluorescence intensity using Image J software, and values were normalized to the control group.

### 2.12 Western Blot

Total proteins were extracted using RIPA lysis buffer (Beyotime, Cat# P0013D) containing PMSF (Biosharp, Cat# BL507A) and protease inhibitor cocktail (Biosharp, Cat# BL612A) (100:1:2). Proteins were separated by SDS‐PAGE and transferred to PVDF membranes (Millipore, Cat# IPVH00010). After blocking with 5% BSA (Servicebio, Cat# GC305010) for 1 h at room temperature, membranes were incubated with primary antibodies at 4°C overnight: rabbit anti‐Drp1 (1:5000, Proteintech, Cat# 1295791‐AP), rabbit anti‐Fis1 (1:5000, Proteintech, Cat# 10956‐1‐AP), rabbit anti‐NME3 (1:1000, Proteintech, Cat# 15136‐1‐AP), rabbit anti‐Mfn1 (1:5000, Proteintech, Cat# 13798‐1‐AP), mouse anti‐Mfn2 (1:5000, Proteintech, Cat# 12186‐1‐AP), mouse anti‐TOM20 (1:5000, Proteintech, Cat# 11802‐1‐AP), rabbit anti‐NDUFB8 (1:1000, Proteintech, Cat# 14749‐1‐AP). Following TBST washes, membranes were incubated with HRP‐conjugated secondary antibodies for 1 h at room temperature. After additional TBST washes, ECL substrate was applied uniformly to the membranes. Chemiluminescent signals were captured using an automated imaging system and semi‐quantitatively analyzed with ImageJ software.

### 2.13 RNA Sequencing and Gene Set Enrichment Analysis (GSEA)

Total RNA was extracted using TRIzol reagent, and its quantity and purity were assessed with Bioanalyzer 2100 and RNA 6000 Nano LabChip Kit. RNA samples with RIN > 7.0 were used for library construction. mRNA was purified from 5 μg total RNA using Dynabeads Oligo (dT) with two rounds of purification. mRNA was fragmented using Magnesium RNA Fragmentation Module, followed by reverse transcription with SuperScript II Reverse Transcriptase to synthesize cDNA. U‐labeled second‐stranded DNA was synthesized using *E. coli* DNA polymerase I, RNase H, and dUTP Solution. Sequencing was performed on an Illumina Novaseq 6000 (2 × 150 bp paired‐end). Gene Set Enrichment Analysis (GSEA) was conducted using GSEA (v4.1.0) and MSigDB to identify enriched KEGG pathways with significant differences, and enrichment scores and *p*‐values were calculated. The relevant bioinformatics analysis is completed by LC‐Bio Technology Co. Ltd.

NME3 expression was analyzed in induced pluripotent stem cell (iPSC)‐derived dopaminergic neurons and midbrain organoids from PD patients using publicly available datasets. Raw data from GSE238129 and GSE278265 were downloaded from the NCBI GEO database, and NME3 expression levels were extracted and analyzed accordingly.

### 2.14 Statistical Analysis

Western blot protein band intensities were quantified using ImageJ, while flow cytometry data were analyzed with FlowJo. All statistical analyses were performed using GraphPad Prism 8.0, with a minimum sample size of *n* ≥ 4 per experimental group. Data were analyzed by Student's *t*‐test, one‐way ANOVA, or two‐way ANOVA as appropriate, and are presented as mean ± S.E.M. Statistical significance was defined as *p* < 0.05.

## 3 Results

### 3.1 NME3 As a Novel Target in the Pathogenesis of PD

**FIGURE 1:**
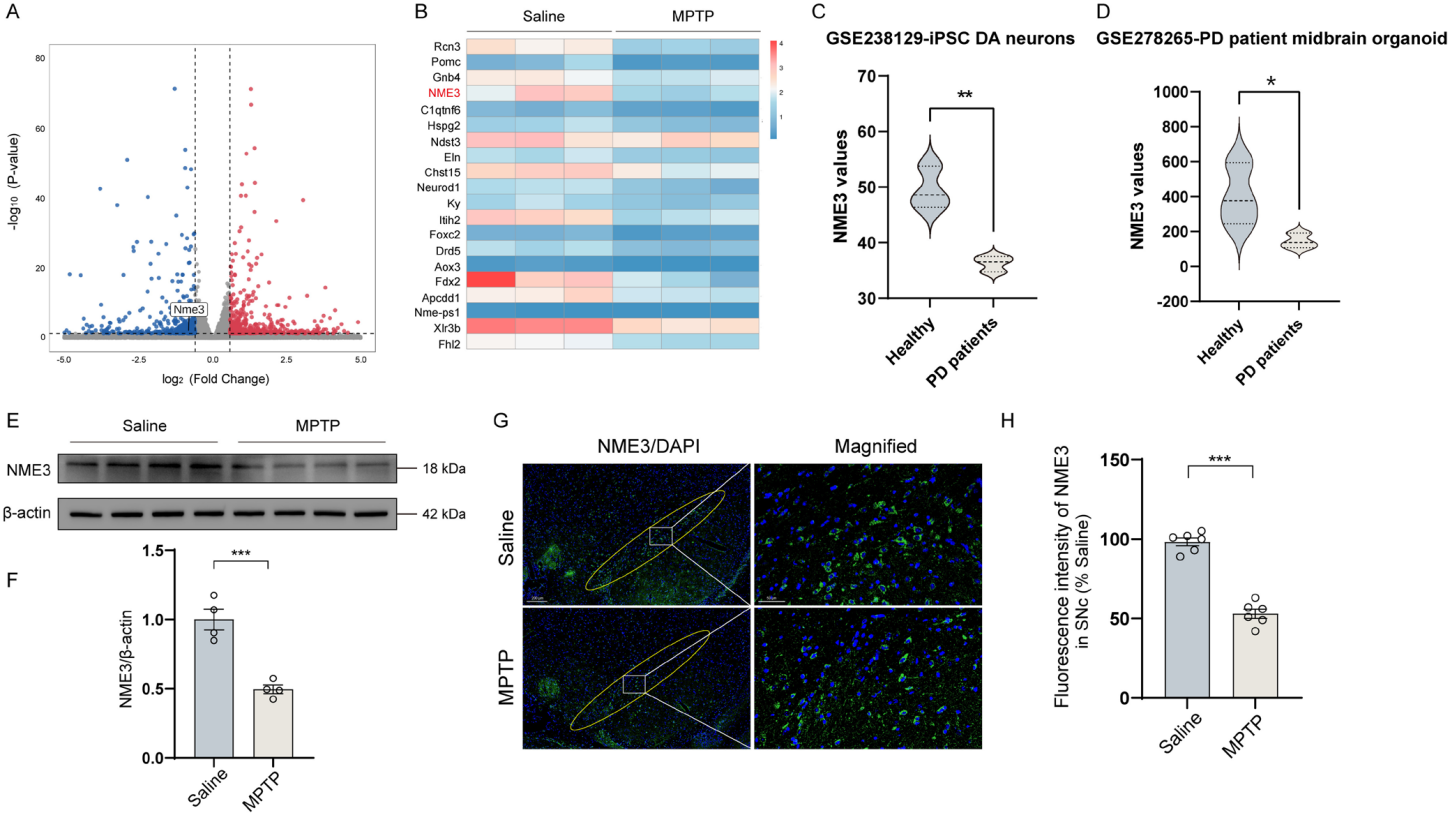
The expression of NME3 was significantly downregulated in PD. (A) Volcano plot illustrates DEGs between Saline and MPTP groups with |log FC| > 1 and *p* < 0.01. (B) Heatmap displays 20 upregulated (red) and downregulated (blue) DEGs between MPTP‐treated and normal mice. (C) Analysis of the expression of NME3 in iPSC‐derived DA neurons using GEO dataset GSE238129. (D) Analysis of the expression of NME3 in PD patient‐derived midbrain organoids in GSE278265. (E) Western blot of NME3 in midbrain tissues collected from Saline‐ and MPTP‐treated mice. (F) Grayscale quantification of NME3 protein levels in the midbrain tissues of Saline‐ and MPTP‐treated mice (*n* = 4 mice per group). (G) Immunofluorescence staining of NME3 (green) in the SNc region of Saline‐ and MPTP‐treated mice. (H) Stereological counts of NME3‐ir cells in the SNc, the sections were taken every six slices in midbrain (*n* = 6 mice per group). Data represent mean ± S.E.M. and analyzed by t‐test. *p < 0.05, **p < 0.01, ***p < 0.001.

Transcriptome sequencing was performed on the midbrain tissues of MPTP‐induced PD model mice. The distribution of differentially expressed genes (DEGs) was shown in a volcano plot (Figure 1A). In the heat map analysis, it was revealed 20 significantly upregulated and downregulated differentially expressed DEGs between MPTP‐treated and normal mice (Figure 1B). Notably, transcriptomic analysis revealed significantly downregulated expression of NME3 in the midbrain of MPTP‐treated mice. Specifically, we observed reduced NME3 expression in iPSC‐derived dopaminergic neurons (GSE278265, Figure 1C) and PD patient‐derived midbrain organoids (GSE238129, Figure 1D). To further validate the sequencing results, western blot analysis demonstrated a 50.9% decrease in NME3 protein levels in the midbrain of MPTP‐induced mice (Figure 1E,F). Consistent with these findings, immunofluorescence analysis revealed significantly suppressed NME3 protein expression in the SNc of MPTP‐treated mice (Figure 1G), showing a 46.1% reduction compared with the saline control group (Figure 1H). These results suggest that NME3 may serve as a critical molecular target regulating PD pathogenesis and disease progression.

### 3.2 NME3 Deficiency Induces PD‐Like Motor Dysfunction and Exacerbates DA Neuron Loss

**FIGURE 2:**
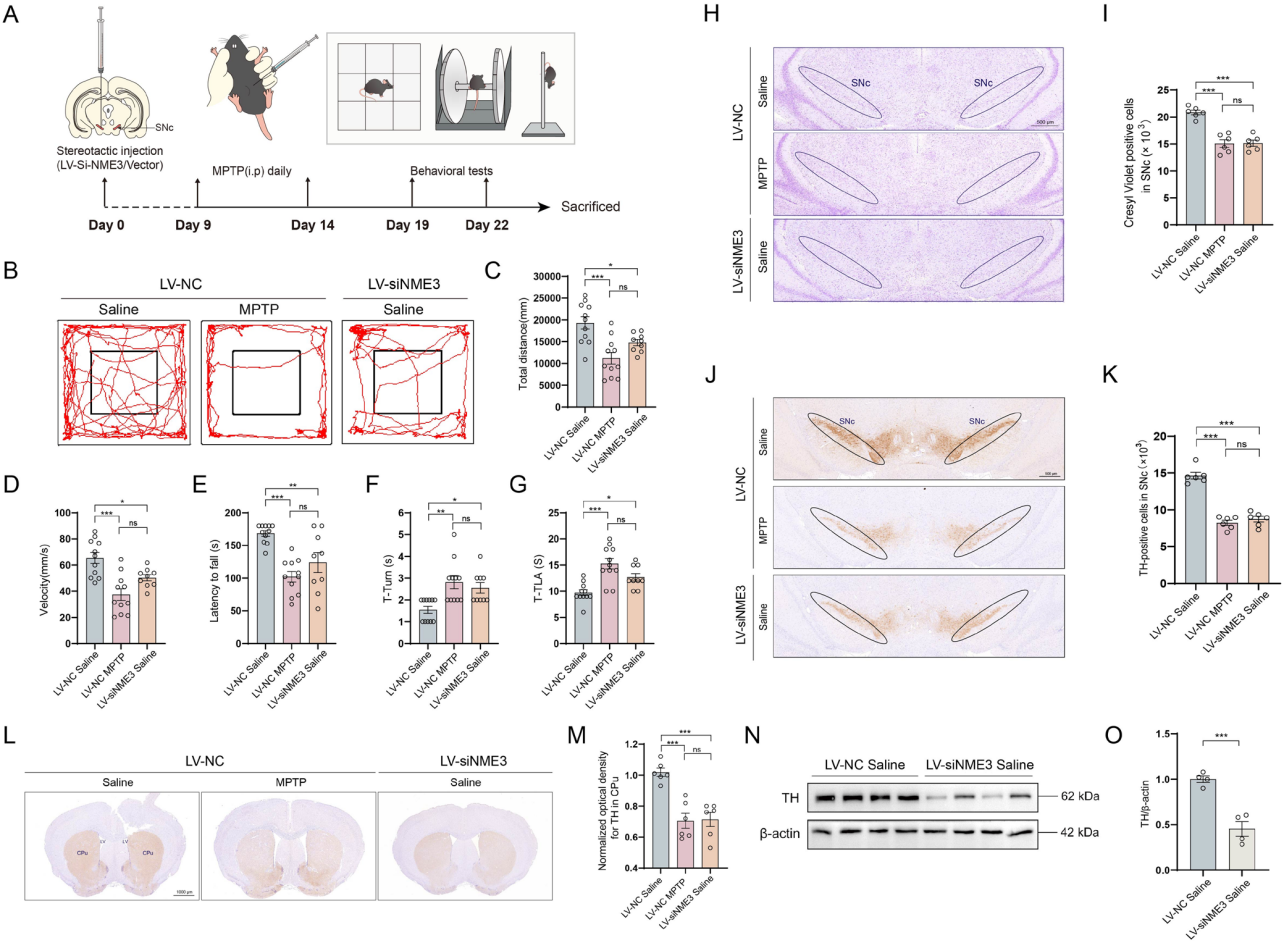
NME3 deficiency induces PD‐like motor dysfunction and DA neuronal loss. (A) Schematic diagram of the mouse model preparation. (B) Movement trajectories of mice in the open field test (*n* = 9–11 mice per group). (C) Total distance traveled by mice in the open field test (*n* = 9–11 mice per group). (D) Movement speed of mice in the open field test (*n* = 9–11 mice per group). (E) Latency to fall in the rotarod test (*n* = 9–11 mice per group). (F) Time to initiate movement until axial rotation in the pole test (*n* = 9–11 mice per group). (G) Time to descend to the base of the pole in the pole test (*n* = 9–11 mice per group). (H) Representative Nissl‐stained images of the SNc region. (I) Quantitative analysis of Nissl‐positive cells (*n* = 6 mice per group). (J) Representative immunohistochemical images of TH‐positive neurons in the SNc. (K) Stereological counting of TH‐positive cells in the SNc (*n* = 6 mice per group). (L) Representative immunohistochemical images of TH‐positive neurons in the CPu. (M) Representative immunohistochemical images of TH‐positive neurons in the CPu (*n* = 6 mice per group). (N) Representative Western blot of TH protein in the midbrain. (O) Quantitative analysis of TH expression (*n* = 4 mice per group). Data represent mean ± S.E.M. and analyzed by t‐test in O and one‐way ANOVA in others. *p < 0.05, **p < 0.01, ***p < 0.001; ns indicates no significant difference.

To investigate the regulatory role of NME3 in PD pathogenesis, we constructed lentivirus‐packaged NME3 knockdown plasmids for in vivo analysis of NME3 deficiency on motor behavior and pathological damage in mice. NME3‐knockdown lentivirus was injected into the SNc of mouse midbrain. One‐week post‐injection, immunohistochemical analysis revealed abundant GFP‐positive cells in brain tissues (Figure S1), confirming precise stereotaxic targeting. In parallel, a subacute MPTP‐induced PD mouse model was established (experimental design shown in Figure 2A). To assess motor coordination, we employed the open field test, rotarod test, and pole test. As shown in Figure 2B–D, NME3 knockdown significantly reduced both total movement distance and velocity in the open field test, mirroring the motor deficits induced by MPTP. Additionally, both NME3 knockdown and MPTP treatment markedly decreased latency to fall in the rotarod test (Figure 2E). The pole test further revealed that NME3 knockdown, similar to MPTP treatment, significantly prolonged the time to initiate body axis reversal (T‐Turn, Figure 2F) and the total descent time (T‐TLA, Figure 2G). These behavioral results collectively demonstrate that NME3 knockdown induces PD‐like motor dysfunction, mirroring the effects of MPTP treatment.

To further elucidate the role of NME3 in neuronal degeneration, Nissl staining was performed on midbrain sections to quantify neuronal populations. Consistent with MPTP‐induced pathology, NME3 knockdown mice exhibited significant neuronal loss in the SNc (Figure 2H,I). Immunohistochemical analysis using tyrosine hydroxylase (TH) staining revealed a 40.3% loss of DA neurons in the SNc (Figure 2J,K) and a 20.8% reduction of TH‐positive neurons in the striatum (Figure 2 L and M). Western blot analysis confirmed that both MPTP treatment and NME3 knockdown significantly downregulated TH expression in midbrain tissues compared with saline controls (Figure 2N,O). Neuroinflammation in the CNS is primarily driven by aberrant activation of immune cells, with microglia and astrocytes serving as pivotal cellular mediators that play central roles in this process [26, 27]. To assess neuroinflammation, immunohistochemical analysis of midbrain sections was performed to detect microglia (labeled with IBA‐1) and astrocytes (labeled with GFAP) (Figure S2). The results showed that NME3 knockdown significantly increased the area of both IBA‐1‐ and GFAP‐positive signals. Taken together, these findings demonstrate that NME3 deficiency in the SNc leads to PD‐like behavioral phenotypes in mice, likely driven by the NME3 knockdown‐induced loss of dopaminergic neurons in the nigrostriatal pathway.

### 3.3 NME3 Deficiency Induces Aberrant Mitochondrial Fission

**FIGURE 3:**
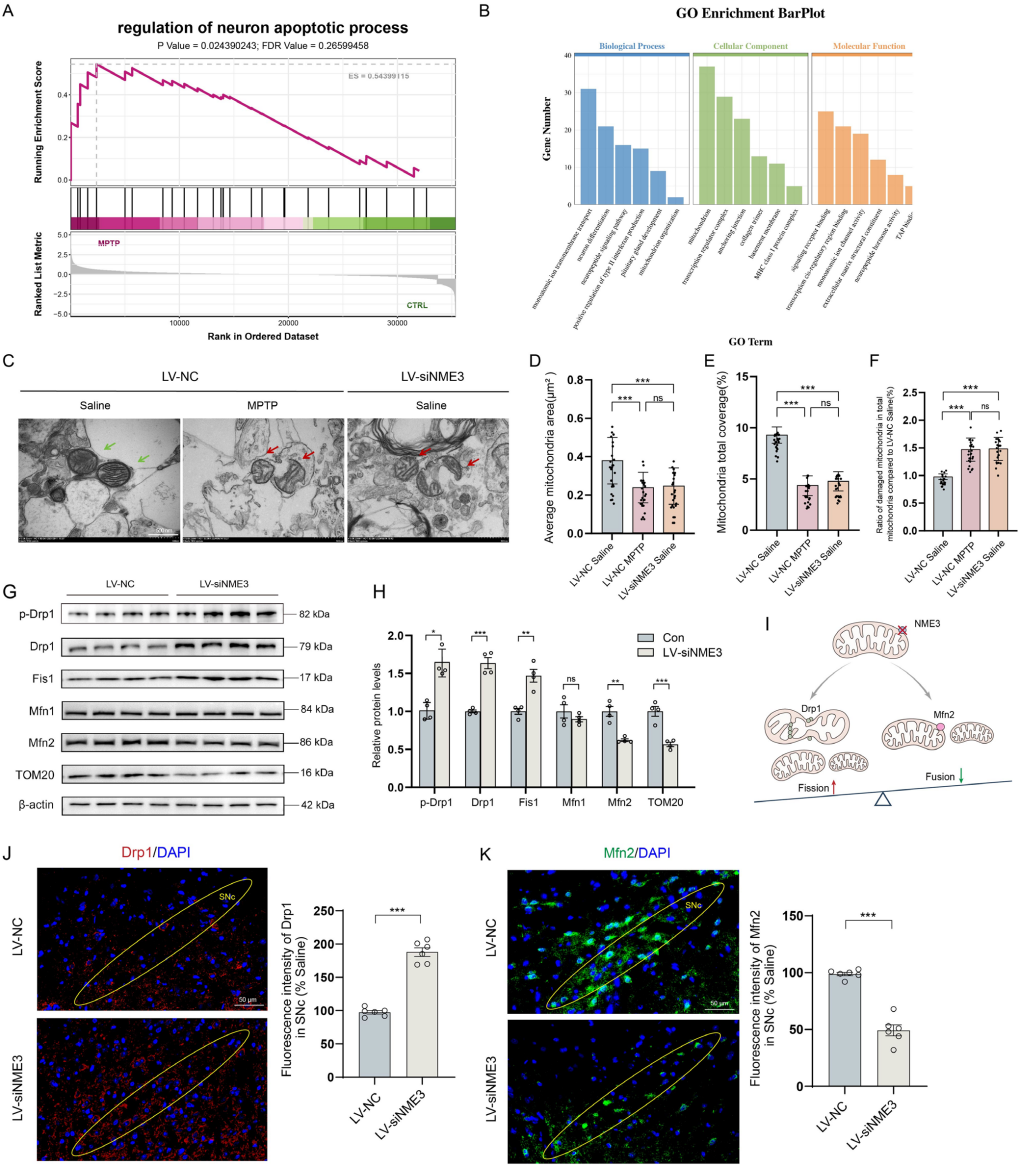
NME3 deficiency induces mitochondrial damage and disrupts mitochondrial dynamics in PD model. (A) Gene set enrichment analysis (GSEA) revealed that NME3 is associated with neuronal apoptosis. (B) GO analysis indicates that NME3 plays an important regulatory role in mitochondria. (C) Morphology of mitochondria in the substantia nigra of LV‐NC and LV‐siNME3 mice treated with MPTP or saline (green arrows indicate normal mitochondria; red arrows indicate mitochondria with abnormal fusion or fission). (D) Individual mitochondrial area. (E) Mitochondrial area as a percentage of cytoplasmic area. (F) The ratio of damaged mitochondria (swelling and fragmentation) in total mitochondria, *n* = 24 from 3 mice per group. (G) Western blot analysis of p‐Drp1, Drp1, Fis1, Mfn1, Mfn2, and TOM20 protein expression in the midbrain tissue of mice. (H) Semi‐quantitative analyses of p‐Drp1, Drp1, Fis1, Mfn1, Mfn2, and TOM20 expression (*n* = 4 mice per group). (I) Proposed model illustrating NME3‐mediated regulation of mitochondrial dynamics. (J) Right: Representative immunofluorescence images of Drp1 (yellow circles mark the SNc region; red fluorescence indicates Drp1; blue fluorescence indicates DAPI). Left: Quantitative analysis of Drp1 fluorescence intensity (*n* = 6 mice per group). (K) Right: Representative immunofluorescence images of Mfn2 (yellow circles mark the SNc region; green fluorescence indicates Mfn2; blue fluorescence indicates DAPI). Left: Quantitative analysis of Mfn2 fluorescence intensity (*n* = 6 mice per group). Data represent mean ± S.E.M. and analyzed by *t*‐test in H, J, and K, and by one‐way ANOVA in D, E, and F. *p < 0.05, **p < 0.01, ***p < 0.001; ns indicates no significant difference.

Previous studies have established that the N‐terminal domain of NME interacts with phosphatidic acid at mitochondrial tips, where it serves as a mitochondrial tether to stimulate Mfn1/2‐mediated fusion, thereby maintaining normal mitochondrial morphology and function [23, 24]. Based on the transcriptome sequencing results, we found that NME3 may play an important role in regulating neuronal apoptosis (Figure 3A) and mitochondrial regulatory functions (Figure 3B). Ultrastructural analysis by TEM revealed significant abnormalities in the mitochondria, accompanied by disrupted fission‐fusion dynamics, in midbrain tissues of both MPTP‐model and NME3‐knockdown mice when compared to saline controls (Figure 3C), as evidenced by reduced mitochondrial area (Figure 3D) and mitochondrial total coverage (Figure 3E) and increased the ratio of damaged mitochondria (Figure 3F). These results demonstrate that the absence of the NME3 induces pathological damage in PD and is associated with the disruption of mitochondrial dynamics balance.

Further Western blot analysis provided additional insight into the regulatory role of NME3 in mitochondrial dynamics‐related proteins during PD pathogenesis. Notably, NME3 knockdown led to an upregulation of fission‐related proteins p‐Drp1/Drp1 and Fis1, while simultaneously downregulating the expression of the fusion protein Mfn2 (Figure 3G,H). Notably, TOM20, an essential mitochondrial protein that maintains protein translocation and function [28], showed significantly decreased levels in the midbrain of NME3‐knockdown mice. This indicates that NME3 may play a role in the dysfunction of mitochondrial transmembrane transport. Based on these findings, we propose that NME3 deficiency contributes to PD pathogenesis by disrupting mitochondrial dynamics homeostasis, manifesting as increased mitochondrial fission and decreased fusion (Figure 3I).

To further validate these observations, we conducted immunofluorescence staining to examine Drp1, a key regulator of mitochondrial fission, and Mfn2, a major mediator of mitochondrial fusion, in the midbrain of the mice. The results demonstrated that NME3 knockdown led to an upregulation of Drp1 expression (Figure 3J) and a concomitant downregulation of Mfn2 levels (Figure 3K) in the SNc. In summary, our findings demonstrate that NME3 deficiency primarily induces mitochondrial morphological abnormalities by disrupting the homeostasis of mitochondrial dynamics. This disruption is characterized by dysregulated Drp1‐Mfn2 expression, leading to excessive mitochondrial fragmentation and the disintegration of the mitochondrial network.

### 3.4 NME3 Knockdown Induces Mitochondrial Dynamics Imbalance in Neurons

**FIGURE 4:**
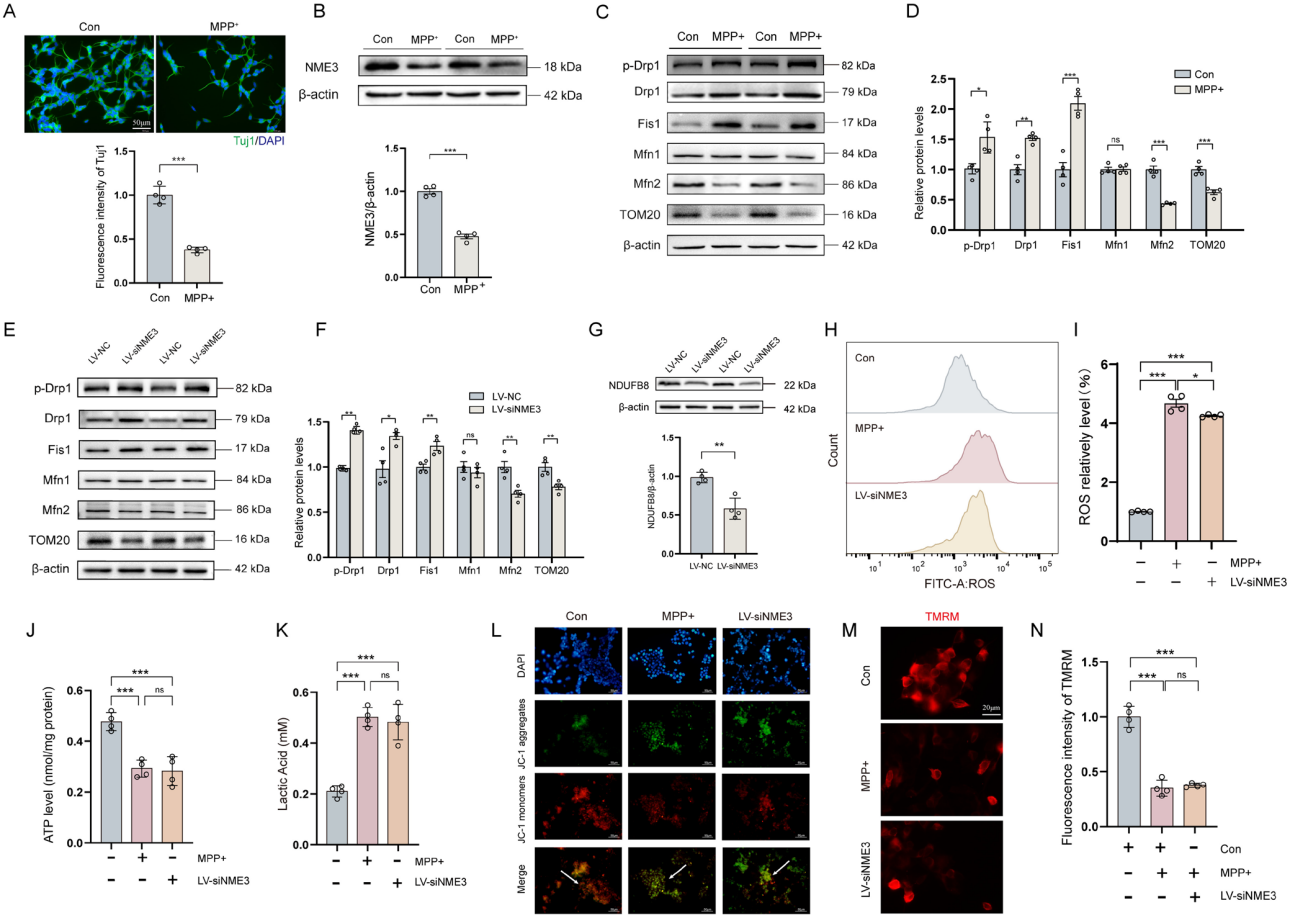
NME3 deficiency induces mitochondrial dynamics imbalance in SH‐SY5Y cells. (A) Upper: The double‐labeling immunofluorescence of Tuj1 and DAPI on differentiated SH‐SY5Y cells. Lower: Analysis of the fluorescence intensity of Tuj1 (*n* = 4 per group). (B) Upper: Representative immunoblot of NME3 protein. Lower: Semi‐quantitative analysis of NME3 expression (*n* = 4 per group). (C) Representative immunoblots of Drp1, Fis1, Mfn1, Mfn2, and TOM20 proteins in SH‐SY5Y cells. (D) Semi‐quantitative analysis of Drp1, Fis1, Mfn1, Mfn2, and TOM20 expression in SH‐SY5Y cells (*n* = 4 per group). (E) Representative immunoblots of p‐Drp1, Drp1, Fis1, Mfn1, Mfn2, and TOM20 proteins in SH‐SY5Y cells. (F) Semi‐quantitative analysis of p‐Drp1, Drp1, Fis1, Mfn1, Mfn2, and TOM20 expression in SH‐SY5Y cells (*n* = 4 per group). (G) Upper: Representative immunoblot of NDUFB8 protein in SH‐SY5Y cells. Lower: Semi‐quantitative analysis of NDUFB8 expression in SH‐SY5Y cells (*n* = 4 per group). (H) ROS levels detected by flow cytometry in SH‐SY5Y cells. (I) Quantitative analysis of ROS levels in SH‐SY5Y cells. (J) Detection of ATP levels in SH‐SY5Y cells (*n* = 4 per group). (K) Detection of lactic acid levels in SH‐SY5Y cells (*n* = 4 per group). (L) Representative fluorescence images of JC‐1 staining (blue fluorescence: DAPI; green fluorescence: JC‐1 aggregates; red fluorescence: JC‐1 monomers; orange fluorescence: Mitochondria with normal membrane potential; green fluorescence: Mitochondria with depolarized membrane potential). (M) TMRM staining (red) in differentiated SH‐SY5Y cells. (N) Analysis of the fluorescence intensity of TMRM in differentiated SH‐SY5Y cells (*n* = 4 per group). Data represent mean ± S.E.M. and analyzed by *t*‐test A‐G, and by one‐way ANOVA in I‐N. *p < 0.05, **p < 0.01, ***p < 0.001; ns indicates no significant difference. Differentiated SH‐SY5Y cells were used in A and M‐N, and undifferentiated SH‐SY5Y cells were used in other experiments.

To validate our previous findings, we established SH‐SY5Y neuronal injury model (Figure S3) in vitro. We used differentiated SH‐SY5Y cells, DA neuron‐like cells, to validate the role of NME3 in MPP^+^‐induced neurotoxicity. Morphologically, MPP^+^ exposure markedly reduced neurite length in differentiated SH‐SY5Y cells, as shown by Tuj1 immunofluorescence staining (Figure 4A). Western blot analysis revealed that MPP^+^ treatment significantly reduced NME3 protein expression in SH‐SY5Y cells (Figure 4B), a result consistent with prior transcriptomic data and observations in PD mouse models.

It is well established that dysregulated mitochondrial dynamics impair neuronal viability and synaptic function, contributing to the pathogenesis of various neurodegenerative disorders, including PD [29, 30]. In this study, we examined mitochondrial dynamics‐related markers in MPP^+^‐treated neurons and found that MPP^+^ treatment led to the upregulation of fission‐related proteins, p‐Drp1/Drp1 and Fis1, while downregulating the fusion protein Mfn2 in SH‐SY5Y cells (Figure 4C,D). These findings indicate that MPP^+^ disrupts the balance of mitochondrial fission and fusion in neurons, further supporting the role of mitochondrial dynamics in PD.

To further elucidate the regulatory role of NME3 in neurons, we also established an NME3‐knockdown model in SH‐SY5Y cells (Figure S4). Interestingly, similar to MPP^+^ treatment, NME3 knockdown induced similar disruptions in mitochondrial dynamics‐related protein expression. Specifically, we observed a significant upregulation of p‐Drp1/Drp1 and Fis1, accompanied by a downregulation of Mfn2, along with reduced expression of TOM20, a key protein involved in mitochondrial function (Figure 4E,F). These patterns of abnormal protein expression mirror that seen in PD pathology, suggesting that NME3 deficiency contributes to mitochondrial fission/fusion imbalance in neurons.

To further characterize mitochondrial ATP production, we assessed NDUFB8, a Complex I subunit in SH‐SY5Y cells. NME3 knockdown reduced NDUFB8 expression (Figure 4G). As well known, mitochondrial dynamics imbalance generates excessive ROS, which triggers cellular oxidative stress and ultimately leads to neuronal damage [31, 32]. To investigate this further, we performed flow cytometry analysis and found that NME3 knockdown impaired mitochondrial electron transport chain function, leading to increased mitochondrial ROS production (Figure 4H,I). Moreover, NME3 deficiency suppressed mitochondrial oxidative phosphorylation (OXPHOS), as evidenced by decreased ATP production and increased lactic acid accumulation; phenotypes were similar to those observed following MPP^+^ treatment (Figure 4J,K). Additionally, assessment of mitochondrial membrane potential revealed that NME3 deficiency enhanced the conversion of JC‐1 aggregates (red fluorescence) to monomers (green fluorescence) in SH‐SY5Y cells (Figure 4L). Consistent with JC‐1 results, TMRM staining revealed that both MPP^+^ treatment and NME3 knockdown led to a significant decrease in mitochondrial membrane potential (Figure 4M,N). These changes are indicative of mitochondrial depolarization, further confirming that NME3 plays a critical role in maintaining mitochondrial dynamics and cellular ATP production.

In short, our results strongly support the hypothesis that NME3 modulates PD‐related pathological mechanisms through the regulation of mitochondrial dynamics homeostasis in neurons. By disrupting the delicate balance of mitochondrial fission and fusion, NME3 deficiency increases ROS generation and promotes neuronal damage, all of which are hallmarks of PD neurodegeneration.

### 3.5 Mdivi‐1 Inhibits NME3 Knockdown‐Induced Mitochondrial Dynamics Imbalance in SH‐SY5Y Cells

**FIGURE 5:**
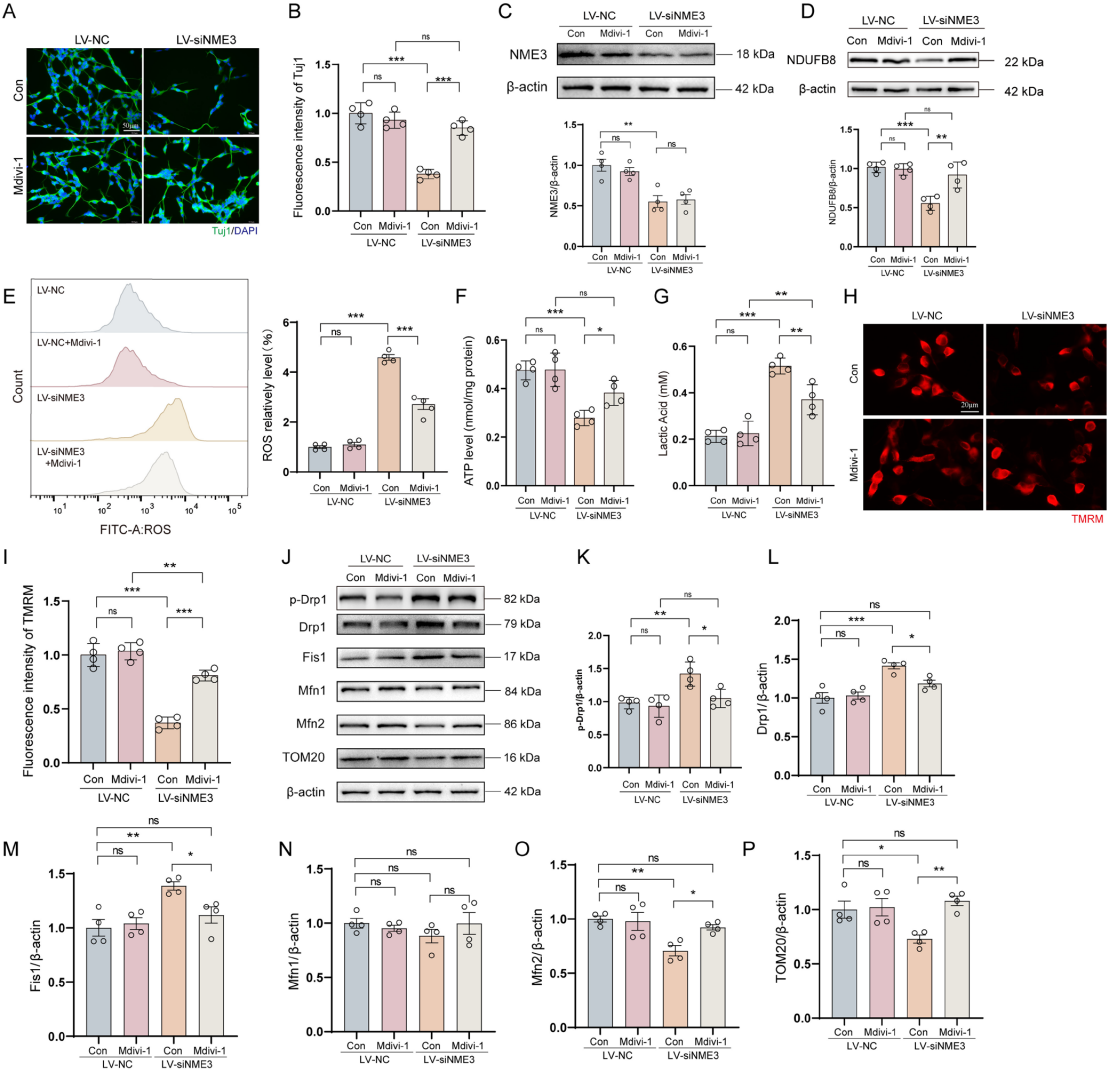
NME3 knockdown induces excessive mitochondrial fission by upregulating Drp1 expression. (A) The double‐labeling immunofluorescence of Tuj1 and DAPI on differentiated SH‐SY5Y cells. (B) Analysis of the fluorescence intensity of Tuj1(*n* = 4 per group). (C) Upper: NME3 protein levels detected by Western blot in SH‐SY5Y cells; Lower: Semi‐quantitative analysis of NME3 expression (*n* = 4 per group). (D) Upper: Representative immunoblot of NDUFB8 protein in SH‐SY5Y cells; Lower: Semi‐quantitative analysis of NDUFB8 expression (*n* = 4 per group). (E) Left: ROS levels detected by flow cytometry in SH‐SY5Y cells; Right: Quantitative analysis of ROS levels (*n* = 4 per group). (F) Detection of ATP levels in SH‐SY5Y cells. (G) Detection of lactic acid levels in SH‐SY5Y cells. (H) TMRM staining (red) in differentiated SH‐SY5Y cells. (I) Analysis of the fluorescence intensity of TMRM (*n* = 4 per group). (J) Protein levels of p‐Drp1, Drp1, Fis1, Mfn1, Mfn2, and TOM20 detected by Western blot in SH‐SY5Y cells. (K–P). Semi‐quantitative analyses of p‐Drp1, Drp1, Fis1, Mfn1, Mfn2, and TOM20 expression (*n* = 4 per group). Data represent mean ± S.E.M. and analyzed by two‐way ANOVA. *p < 0.05, **p < 0.01, ***p < 0.001; ns indicates no significant difference. Differentiated SH‐SY5Y cells were used in A and H‐I, and undifferentiated SH‐SY5Y cells were used in other experiments.

Mitochondrial fission inhibitor 1 (Mdivi‐1) suppresses mitochondrial fission by inhibiting Drp1 activation, primarily through modulation of its GTPase activity [33]. Previous studies have demonstrated that downregulation of NME3 expression results in increased mitochondrial fission and reduced fusion in neurons. To further investigate the role of NME3 in regulating mitochondrial dynamics, this study employed an in vitro approach utilizing Mdivi‐1 treatment. First, we determined the optimal concentration of Mdivi‐1, which was found to be 10 μM based on cell viability assays (Figure S5). In differentiated SH‐SY5Y cells, we found that NME3 knockdown decreased neurite length, similar to MPP^+^ treatment, whereas Mdivi‐1 treatment effectively rescued this phenotype (Figure 5A,B). Western blot analysis revealed that Mdivi‐1 treatment did not affect NME3 protein expression (Figure 5C), suggesting that the inhibitor specifically impacts mitochondrial dynamics without altering NME3 levels.

However, Mdivi‐1 treatment restored NDUFB8 expression (Figure 5D), and flow cytometry analysis demonstrated that Mdivi‐1 effectively reversed the increased ROS release induced by NME3 knockdown (Figure 5E). We also found that Mdivi‐1 treatment rescued the decreased ATP and increased lactic acid induced by NME3 knockdown (Figure 5F,G). TMRM staining further revealed that Mdivi‐1 treatment preserved mitochondrial membrane potential in the absence of NME3 (Figure 5H,I). Further examination of mitochondrial dynamics‐related proteins revealed that Mdivi‐1 treatment did not significantly affect the expression of mitochondrial‐associated proteins in the LV‐NC group. In contrast, in the NME3 knockdown group, Mdivi‐1 treatment significantly reversed the altered expression of mitochondrial fission and fusion proteins. Specifically, Mdivi‐1 treatment restored p‐Drp1/Drp1 and Mfn2 levels to near‐normal values and increased TOM20 expression, while Mfn1 levels remained unchanged across all treatment groups (Figure 5J–P).

These findings demonstrate that NME3 regulates neuronal mitochondrial dynamics homeostasis through the Drp1‐Mfn2 pathway. The increased TOM20 expression further suggests that Mdivi‐1 may restore mitochondrial outer membrane protein transport by suppressing excessive mitochondrial fission. Collectively, these results underscore that NME3 deficiency in neurons exacerbates mitochondrial fission by enhancing Drp1 expression, thereby disrupting mitochondrial homeostasis and contributing to DA neuronal injury.

### 3.6 Neuronal NME3 Overexpression Rescues MPP ^+^‐Induced Imbalance of Mitochondrial Dynamics

**FIGURE 6:**
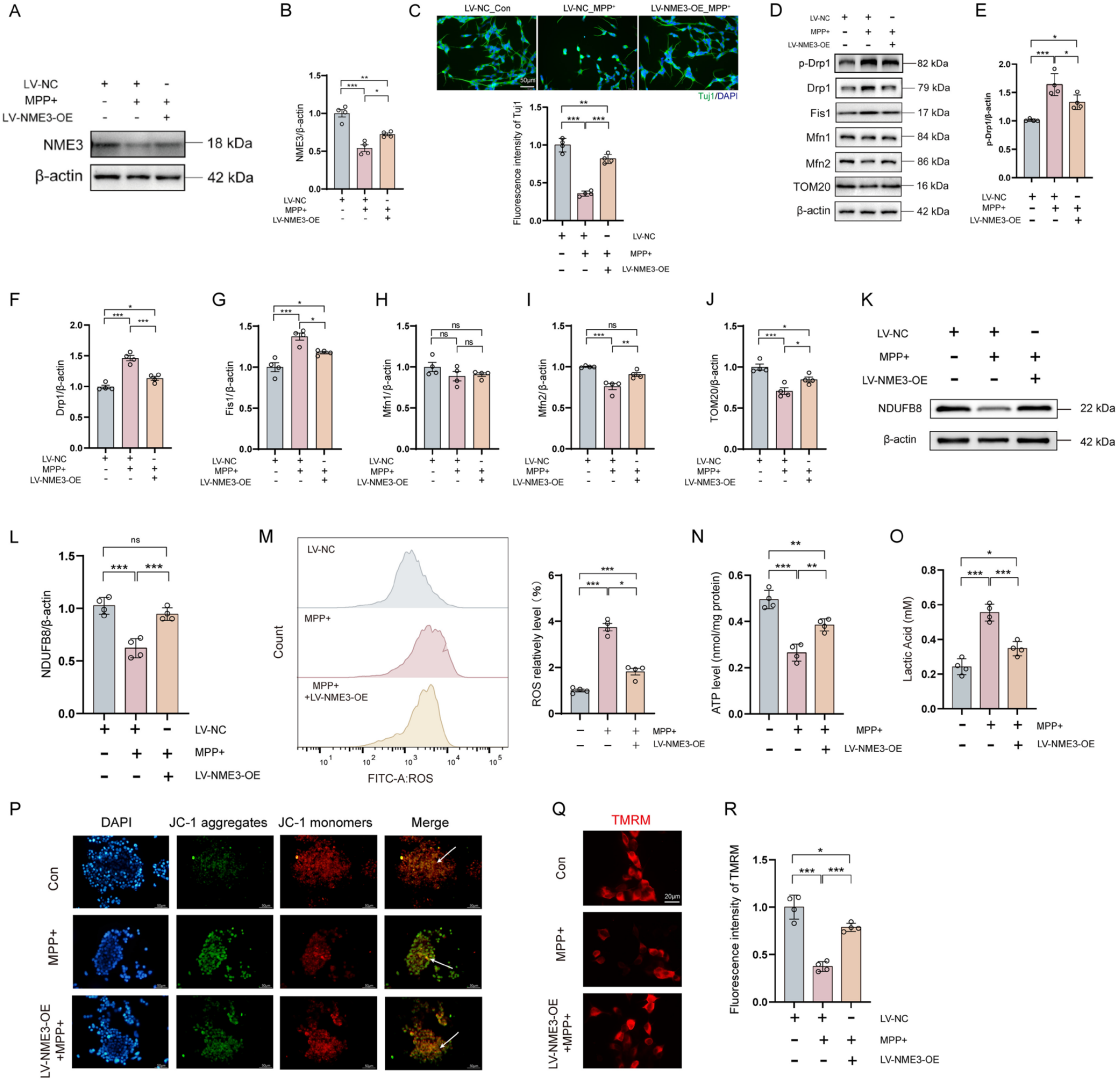
Neuronal NME3 overexpression rescues MPP^+^‐induced mitochondrial dynamics imbalance in SH‐SY5Y cells. (A) Representative immunoblot of NME3 protein in SH‐SY5Y cells. (B) Semi‐quantitative analysis of NME3 expression (*n* = 4 per group). (C) Upper: The double‐labeling immunofluorescence of Tuj1 and DAPI on differentiated SH‐SY5Y cells. Lower: Analysis of the fluorescence intensity of Tuj1 (*n* = 4 per group). (D) Representative immunoblots of p‐Drp1, Drp1, Fis1, Mfn1, Mfn2, and TOM20 proteins in SH‐SY5Y cells. (E‐J) Quantitative analysis of p‐Drp1, Drp1, Fis1, Mfn1, Mfn2, and TOM20 expression (*n* = 4 per group). (K) Representative immunoblot of NDUFB8 protein in SH‐SY5Y cells. (L) Semi‐quantitative analysis of NDUFB8 expression (*n* = 4 per group). (M) Left: ROS levels detected by flow cytometry in SH‐SY5Y cells. Right:Quantitative analysis of ROS levels (*n* = 4 per group). (N) Detection of ATP levels in SH‐SY5Y cells (*n* = 4 per group). (O) Detection of lactic acid levels in SH‐SY5Y cells (*n* = 4 per group). (P) Representative fluorescence images of JC‐1 staining (blue: DAPI; green: JC‐1 aggregates; red: JC‐1 monomers; orange: Mitochondria with normal membrane potential; green: Mitochondria with depolarized membrane potential). (Q) TMRM staining (red) in differentiated SH‐SY5Y cells. (R) Analysis of the fluorescence intensity of TMRM (*n* = 4 per group). Data represent mean ± S.E.M. and analyzed by one‐way ANOVA. *p < 0.05, **p < 0.01, ***p < 0.001; ns indicates no significant difference. Differentiated SH‐SY5Y cells were used in C and Q‐R, and undifferentiated SH‐SY5Y cells were used in other experiments.

Although Mdivi‐1 has been shown to regulate mitochondrial dynamics and alleviate neurodegenerative pathologies in preclinical research, its direct clinical translation is limited by insufficient target specificity, suboptimal pharmacokinetics, and potential toxicity [34]. In contrast, the small molecular weight and high expression specificity of NME3 make it a promising candidate as a small‐molecule protein modulator for future therapeutic interventions in PD. To further elucidate the precise regulatory role of NME3 in PD, we constructed lentivirus‐packaged NME3 over‐expression plasmids. Transfection of SH‐SY5Y cells with overexpression lentivirus (Figure S6) successfully reversed the MPP^+^‐induced suppression of NME3 expression. Restoration of NME3 expression, from 54% to 72% (Figure 6A,B). In differentiated SH‐SY5Y cells, NME3 overexpression repaired the decreased neurite length by MPP^+^ treatment (Figure 6C), which was accompanied by an effective reversal of the MPP^+^‐induced upregulation of fission‐related proteins p‐Drp1/Drp1 and Fis1, as well as suppression of the fusion‐related protein Mfn2 (Figure 6D–J).

Moreover, overexpression of NME3 significantly up‐regulated the expression of MPP^+^‐inhibited NDUFB8 (Figure 6K,L), and suppressed MPP^+^‐induced abnormal accumulation of ROS in neuronal cells (Figure 6M), along with the reversal of decreased ATP (Figure 6N) and increased lactic acid production (Figure 6O). Representative image analysis of mitochondrial membrane potential revealed that restored NME3 expression reversed the conversion of JC‐1 aggregates (red fluorescence) to monomers (green fluorescence) (Figure 6P), similar to the findings in TMRM staining (Figure 6Q,R), demonstrating that NME3 overexpression effectively counteracts MPP^+^‐induced mitochondrial depolarization.

These findings further elucidate that NME3 participates in PD pathogenesis by regulating neuronal mitochondrial dynamics homeostasis. These molecular alterations suggest that NME3, as a small protein, may effectively mimic the biological effects of Mdivi‐1 by targeting the Drp1–Mfn2 pathway, thereby preserving the balance between mitochondrial fission and fusion.

### 3.7 NME3 Overexpression Ameliorates MPTP‐Induced PD‐Like Symptoms in Mice

**FIGURE 7:**
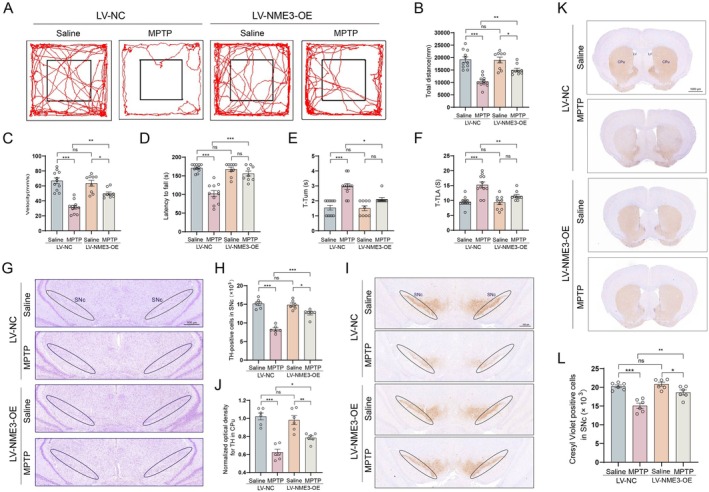
Restoration of NME3 expression ameliorates MPTP‐induced motor dysfunction and DA neuronal loss. (A) Representative movement trajectories of mice in the open field test (*n* = 9–11 mice per group). (B) Total distance traveled in the open field test (*n* = 9–11 mice per group). (C) Movement speed in the open field test (*n* = 9–11 mice per group). (D) Latency to fall in the rotarod test (*n* = 9–11 mice per group). (E) Time to axial rotation in the pole test (*n* = 9–11 mice per group). (F) Time to descend in the pole test (*n* = 9–11 mice per group). (G) Representative Nissl‐stained images of the SNc brain slices. (H) Quantitative analysis of Nissl‐positive cells (*n* = 6 mice per group). (I) Representative immunohistochemical images of TH‐positive neurons in the SNc brain slices. (J) Stereological counting of TH‐positive neurons in the SNc (*n* = 6 mice per group). (K) Representative immunohistochemical images of TH‐positive neuronal fibers in the CPu sections. (L) Quantitative analysis of TH‐positive neuronal fibers in the CPu (*n* = 6 mice per group). Data represent mean ± S.E.M. and analyzed by two‐way ANOVA. *p < 0.05, **p < 0.01, ***p < 0.001; ns indicates no significant difference.

To further investigate whether the restoration of NME3 expression could ameliorate PD pathological phenotypes in vivo, we administered NME3‐overexpressing lentivirus to MPTP‐induced PD model mice. Behavioral tests demonstrated that NME3 overexpression significantly ameliorated MPTP‐induced motor dysfunction in PD model mice, compared to the negative control (NC) group. Specifically, NME3 overexpression improved several aspects of motor performance, including: a decreased movement distance (Figure 7A,B), reduced movement speed (Figure 7A,C) during the open field test, a reduction in latency to fall in the rotarod test (Figure 7D), and improved pole test performance, characterized by decreased time to axial turn and decreased descent time to the base (Figure 7E,F).

Moreover, compared to the NC group, NME3 overexpression notably attenuated MPTP‐induced neuronal loss in the SNc (Figure 7G,H), as well as the reduction of TH‐positive neurons in both the SNc (Figure 7I,J) and CPu (Figure 7K,L) regions. These findings suggest that NME3 may function as a small‐molecule protein exerting neuroprotective effects in PD pathogenesis.

### 3.8 NME3 Overexpression Rescues MPTP‐Induced Mitochondrial Fission/Fusion Imbalance

**FIGURE 8:**
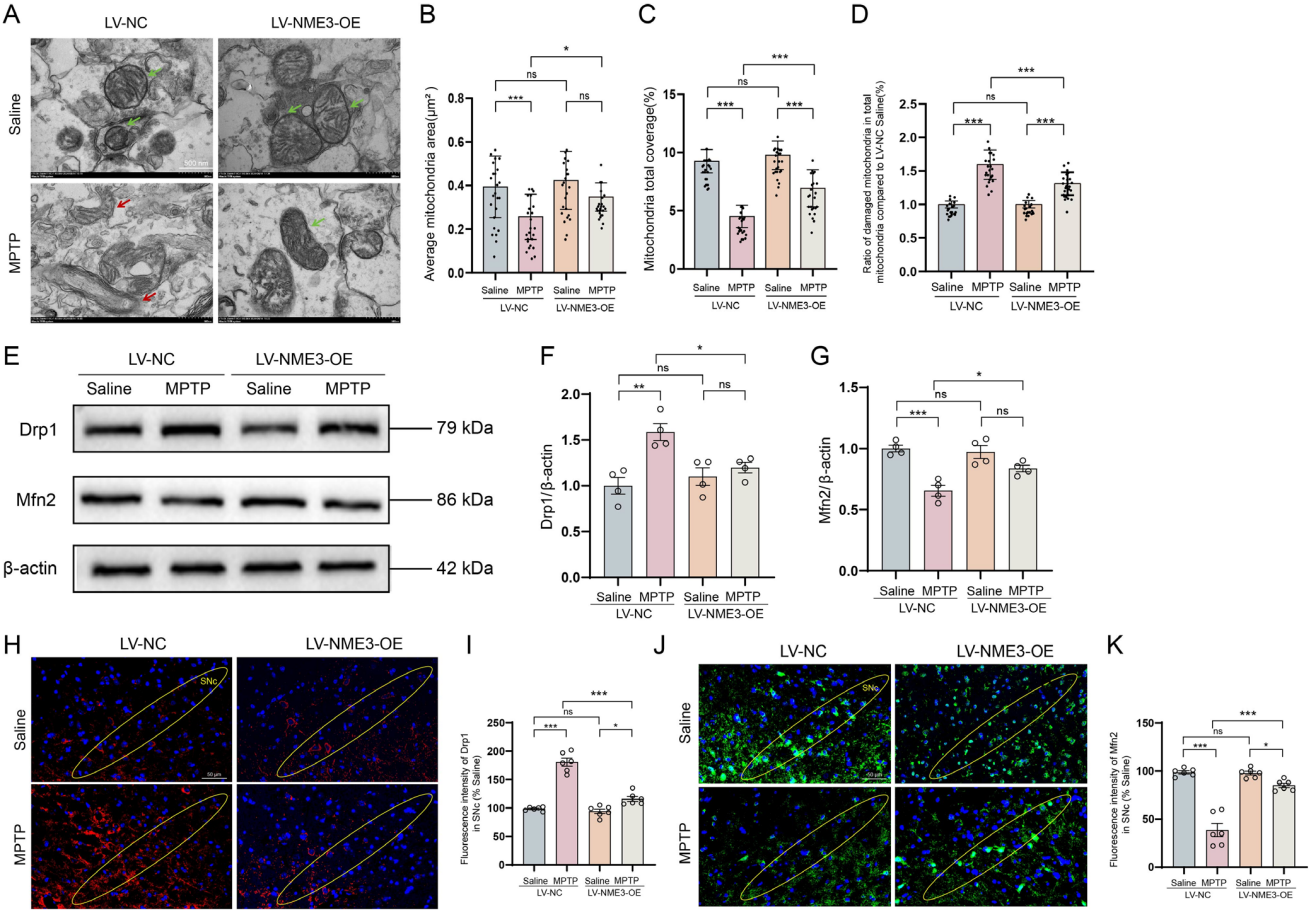
Restoration of NME3 expression ameliorates MPTP‐induced mitochondrial damage and mitochondrial dynamics imbalance. (A) Transmission electron micrographs of midbrain mitochondria (green arrows: Normal mitochondria; red arrows: Mitochondria with abnormal fusion/fission). (B) Individual mitochondrial area. (C) Mitochondrial area as a percentage of cytoplasmic area. (D) The ratio of damaged mitochondria (swelling and fragmentation) in total mitochondria (*n* = 24 from 3 mice per group). (E) Representative immunoblots of Drp1, Fis1, Mfn1, Mfn2, and TOM20 proteins in the midbrain tissue. (F and G) Semi‐quantitative analysis of Drp1 and Mfn2 expression (*n* = 4 mice per group). (H) Representative immunofluorescence images of Drp1 (red: Drp1; blue: DAPI) in the SNc sections. (I) Quantitative analysis of Drp1 fluorescence intensity (*n* = 6 mice per group). (J) Representative immunofluorescence images of Mfn2 (green: Mfn2; blue: DAPI) in the SNc sections. (K) Quantitative analysis of Mfn2 fluorescence intensity (*n* = 6 mice per group). Data represent mean ± S.E.M. and analyzed by two‐way ANOVA. *p < 0.05, **p < 0.01, ***p < 0.001; ns indicates no significant difference.

To further elucidate the effects of NME3 on mitochondrial structure and function in vivo. The results of TEM showed that, compared with the NC group, overexpression of NME3 repaired the abnormal mitochondrial fission and reversed the abnormal mitochondrial morphology induced by MPTP (Figure 8A), as evidenced by increased individual mitochondrial area (Figure 8B) and mitochondrial total coverage (Figure 8C), and decreased ratio of damaged mitochondria (Figure 8D). Western blot analysis demonstrated that, compared to the NC group, NME3 overexpression significantly attenuated MPTP‐induced upregulation of mitochondrial fission regulators Drp1 (Figure 8E,F) and restored the expression of fusion regulator Mfn2 (Figure 8E,G) in mouse midbrain tissues. This study further performed immunofluorescence staining of Drp1 and Mfn2, revealing that NME3 overexpression in the mouse midbrain SNc significantly suppressed both MPTP‐induced upregulation of Drp1 expression (Figure 8H,I) and downregulation of Mfn2 expression (Figure 8J,K). These results demonstrated that NME3 maintains mitochondrial structural integrity by suppressing excessive fission and promoting fusion‐mediated repair, thereby rescuing mitochondrial dynamics imbalance. These reveal a novel mechanism whereby NME3 regulates the Drp1/Mfn2 axis to participate in mitochondrial homeostasis maintenance in PD.

## 4 Discussion

In this study, we uncover a novel role of NME3 in PD, driven by its regulation of mitochondrial dynamics homeostasis through the Drp1‐Mfn2 pathway. Specifically, NME3 is a mitochondrial‐stabilizing protein localized to the outer mitochondrial membrane, and its deficiency induces PD‐like dysfunction and DA neuronal degeneration. Concurrently, NME3 deficiency induces abnormal mitochondrial morphology and dysregulated expression of key mitochondrial dynamics regulators, Drp1 and Mfn2, consequently triggering an imbalance in mitochondrial fission–fusion homeostasis. Importantly, we demonstrate that NME3 overexpression reverses MPTP‐induced motor dysfunction and DA neuronal loss in vivo, as well as mitigates MPP^+^‐mediated neuronal damage and excessive mitochondrial fission. By restoring mitochondrial dynamics homeostasis, NME3 shows promise as a potential therapeutic target for PD.

Imbalanced mitochondrial dynamics is a common pathological feature of neurodegenerative diseases [18]. In PD, although existing studies have demonstrated a close association between abnormal mitochondrial fission/fusion and DA neurodegeneration, the key molecular mechanisms remain incompletely elucidated [19, 35]. This study is the first to propose the protective role and molecular mechanism of NME3 in the pathological process of PD. Both transcriptomic analysis and experimental validation in this study demonstrated a significant downregulation of NME3 expression in the SNc of PD mouse models. More importantly, NME3 deficiency exacerbates DA neuronal death and leads to mitochondrial network fragmentation, characterized by excessive fission and impaired fusion. These findings were further validated in vitro, where NME3 knockdown in neuronal cells similarly resulted in mitochondrial dynamic imbalance and excessive ROS production. Notably, administration of the mitochondrial fission inhibitor Mdivi‐1 effectively ameliorated both mitochondrial dynamics imbalance and neurodegenerative phenotypes in the PD model. These findings demonstrate that NME3 exerts neuroprotective effects by maintaining mitochondrial homeostasis through regulation of the Drp1‐Mfn2 pathway.

Glial cells interact with neurons by regulating the neuronal microenvironment, providing metabolic support, and mediating immune responses, collectively maintaining nervous system homeostasis [26]. Astrocytes provide metabolic support to neurons, while microglia, as the immune cells of the brain, participate in inflammatory responses and cellular debris clearance [36, 37]. A key finding of this study is the abnormal proliferation and activation of glial cells in the SNc region of NME3‐knockdown mice (Figure S2). This phenomenon may arise through a dual pathway: on one hand, NME3 deficiency‐induced imbalance of mitochondrial dynamics prompts neurons to release damage‐associated molecular patterns (DAMPs); on the other hand, excessive mitochondrial ROS production further activates inflammatory responses in glial cells. This vicious cycle likely serves as a critical accelerator for DA neuronal degeneration.

TOM20, an essential outer mitochondrial membrane protein, maintains mitochondrial functional homeostasis by recognizing precursor proteins, regulating transmembrane transport, and participating in quality control processes [38]. The reduced expression of TOM20 observed following NME3 knockdown may arise through two potential pathways: First, the mitochondrial dynamics instability induced by NME3 deficiency could lead to mitochondrial fragmentation, thereby diminishing membrane localization of TOM20. Alternatively, excessive ROS accumulation triggered by NME3 knockdown might accelerate TOM20 degradation through activation of proteasome degradation or mitophagy pathways. Our future research will focus on verifying the critical role of TOM20 in PD pathogenesis by either restoring its expression or inhibiting ROS production.

In the MPTP‐induced PD mouse model, NME3 overexpression resulted in significant behavioral improvements and reduced DA neuronal loss. At the molecular level, NME3 overexpression effectively suppressed excessive mitochondrial fission and promoted mitochondrial network reconstruction. These findings not only validate the neuroprotective role of NME3 in PD, but also suggest that targeting the NME3‐mitochondrial pathway may represent a novel therapeutic strategy for PD. Complementary models, such as α‐synuclein transgenic mice or α‐synuclein preformed fibril‐induced PD models, could be employed in future studies to further validate the role of NME3 in regulating mitochondrial dynamics and its therapeutic potential.

In summary, our study provides the first demonstration that NME3 protects DA neurons against PD‐associated damage by maintaining mitochondrial homeostasis through regulation of the Drp1‐Mfn2 axis. This discovery not only deepens our understanding of PD pathogenesis but also provides a novel molecular target for developing disease‐modifying therapies based on mitochondrial homeostasis regulation.

## Author Contributions

**Chen Qiao:** writing – original draft, validation, resources, investigation, funding acquisition, data curation. **Xiang‐Qi Hu:** methodology, software, data processing. **Shen‐Han Xu, Meng‐Fan Yao:** formal analysis, methodology, writing – original draft. **Jun‐Peng Liu:** investigation, project administration. **Lei Cao:** writing – review and editing, validation, investigation, funding acquisition.

## Funding

The work reported herein was supported by the grants from the Natural Science Foundation of Jiangsu Province (BK20241858), the National Natural Science Foundation of China (82204357, 81803505), Medical Research Project of Jiangsu Provincial Health Commission (M2022071), Jiangsu Research Hospital Association for Precision Medication (JY202232), Basic Research Foundation of Zhenjiang (JC2024024).

## Ethics Statement

The experimental protocol was reviewed and approved by the Institutional Animal Care and Use Committee of Nanjing Medical University (IACUC No. 2008067).

## Conflicts of Interest

The authors declare no conflicts of interest.

## Supporting information


**Figure S1:** In vivo establishment of NME3 knockdown/overexpression mouse models. (A) Schematic diagram of successful stereotactic intracerebral injection of NME3 knockdown lentivirus. (B) Representative immunoblot of NME3 protein in the midbrain tissue of mice. (C) Semi‐quantitative analysis of NME3 expression (*n* = 4 mice per group). (D) Schematic diagram of stereotactic intracerebral injection of NME3 overexpression lentivirus. (E) Representative immunoblot of NME3 protein in the midbrain tissue of mice. (F) Quantitative analysis of NME3 expression (*n* = 4 mice per group). Data represent mean ± S.E.M. and analyzed by *t*‐test. *p < 0.05, **p < 0.01.
**Figure S2:** NME3 knockdown induces abnormal proliferation and activation of glial cells. (A) Representative immunofluorescence images of IBA1 labeling (red) in the SNc region of mice. (B) Quantitative analysis of IBA1 fluorescence intensity (*n* = 6 mice per group). (C) Representative immunofluorescence images of GFAP labeling (purple). (D) Quantitative analysis of GFAP fluorescence intensity (*n* = 6 mice per group). Data represent mean ± S.E.M. and analyzed by one‐way ANOVA. **p < 0.01, ***p < 0.001; ns indicates no significant difference.
**Figure S3:** Establishment of MPP^+^‐induced PD cell model and optimization of MPP^+^ concentration and treatment duration. (A) CCK‐8 assay assessing SH‐SY5Y cell viability under different MPP^+^ concentrations and treatment durations (*n* = 4 per group). The optimal condition for PD model induction was determined to be 250 μM MPP^+^ for 48 h, which was subsequently used for follow‐up cellular experiments. (B) Representative morphological images of control and MPP^+^‐treated model cells. Data represent mean ± S.E.M. and analyzed by one‐way ANOVA. ***p < 0.001; ns indicates no significant difference.
**Figure S4:** LV‐NME3‐siRNA inhibits NME3 expression in SH‐SY5Y cells. (A) GFP‐positive cells (green) demonstrate lentiviral transfection efficiency, with the optimal titer determined to be 2.14 × 10^8^ TU/mL for SH‐SY5Y cell transduction. (B) Representative immunoblot of NME3 protein. LV‐NME3‐siRNA (Y22378) was selected as the optimal lentiviral vector for subsequent experiments. (C) Semi‐quantitative analysis of NME3 expression (*n* = 4 per group). Data represent mean ± S.E.M. and analyzed by one‐way ANOVA. *p < 0.05, ***p < 0.001.
**Figure S5:** CCK8 assay evaluates the effects of different Mdivi‐1 concentrations on SH‐SY5Y cell viability. Data represent mean ± S.E.M. and analyzed by one‐way ANOVA; ns indicates no significant difference.
**Figure S6:** Lentivirus‐packaged LV‐NME3‐OE upregulates NME3 expression in SH‐SY5Y cells. (A) Determination of optimal lentiviral titer for SH‐SY5Y cell transduction with GFP (green fluorescent protein) indicating transduction efficiency. (B) Representative immunoblot of NME3 protein levels. (C) Quantitative analysis of NME3 expression (*n* = 4 per group). Data represent mean ± S.E.M. and analyzed by *t*‐test. **p < 0.01.
**Figure S7:** The untreated control group did not affect the expression of Drp1 or Mfn2 expression in vivo and in vitro. (A) Western blot analyses of Drp1 and Mfn2 protein expression in midbrain; Mock, the untreated control group; LV‐NC, the negative control virus group. (B and C) Semi‐quantitative analysis of Drp1 and Mfn2 expression (*n* = 4 mice per group). (D) Western blot analysis of Drp1 and Mfn2 protein expression in SH‐SY5Y; Mock, the untreated control group; LV‐NC, the negative control virus group. (E and F) Semi‐quantitative analysis of Drp1 and Mfn2 expression (*n* = 4 mice per group). Data represent mean ± S.E.M. and analyzed by *t*‐test, ns indicates no significant difference.

## Data Availability

The data that support the findings of this study are available from the corresponding author upon reasonable request.
