# Supplementary material for: Targeting NME3 to Restore Mitochondrial Fission‐Fusion Balance Defines a Novel Disease‐Modifying Strategy for Parkinson's Disease
Source: CNS Neurosci Ther. 2026 Mar 9;32(3):e70822. doi: 10.1002/cns.70822 (PMC12971606; doi:10.1002/cns.70822)
Supplement: Supplementary file 1 — Figure S1: In vivo establishment of NME3 knockdown/overexpression mouse models. (A) Schematic diagram of successful stereotactic intracerebral injection of NME3 knockdown lentivirus. (B) Representative immunoblot of NME3 protein in the midbrain tissue of mice. (C) Semi‐quantitative analysis of NME3 expression (n = 4 mice per group). (D) Schematic diagram of stereotactic intracerebral injection of NME3 overexpression lentivirus. (E) Representative immunoblot of NME3 protein in the midbrain tissue of mice. (F) Quantitative analysis of NME3 expression (n = 4 mice per group). Data represent mean ± S.E.M. and analyzed by t‐test. *p < 0.05, **p < 0.01. Figure S2: NME3 knockdown induces abnormal proliferation and activation of glial cells. (A) Representative immunofluorescence images of IBA1 labeling (red) in the SNc region of mice. (B) Quantitative analysis of IBA1 fluorescence intensity (n = 6 mice per group). (C) Representative immunofluorescence images of GFAP labeling (purple). (D) Quantitative analysis of GFAP fluorescence intensity (n = 6 mice per group). Data represent mean ± S.E.M. and analyzed by one‐way ANOVA. **p < 0.01, ***p < 0.001; ns indicates no significant difference. Figure S3: Establishment of MPP+‐induced PD cell model and optimization of MPP+ concentration and treatment duration. (A) CCK‐8 assay assessing SH‐SY5Y cell viability under different MPP+ concentrations and treatment durations (n = 4 per group). The optimal condition for PD model induction was determined to be 250 μM MPP+ for 48 h, which was subsequently used for follow‐up cellular experiments. (B) Representative morphological images of control and MPP+‐treated model cells. Data represent mean ± S.E.M. and analyzed by one‐way ANOVA. ***p < 0.001; ns indicates no significant difference. Figure S4: LV‐NME3‐siRNA inhibits NME3 expression in SH‐SY5Y cells. (A) GFP‐positive cells (green) demonstrate lentiviral transfection efficiency, with the optimal titer determined to be 2.14 × 10^8^ TU/mL for S [file CNS-32-e70822-s001.docx]

**Supplementary Data**

**Targeting NME3 to restore mitochondrial fission-fusion balance defines a novel disease-modifying strategy for Parkinson's disease**

Chen Qiao^1,2#^, Xiang-Qi Hu^1,2#^, Shen-Han, Xu^3^, Meng-Fan Yao^1,2^, Jun-Peng Liu^1^, Lei Cao^3*^

^1^Department of Clinical Pharmacy, Affiliated Hospital of Jiangsu University, Zhenjiang, Jiangsu 212001, China

^2^College of Pharmacy, Jiangsu University, Zhenjiang, Jiangsu 212013, China

^3^Jiangsu Key Laboratory of Neurodegeneration, Department of Pharmacology, Nanjing Medical University, Nanjing, 211166, China

^#^These authors contributed equally to this work.

^*^Correspondence:

Lei Cao, Ph.D.

Department of Pharmacology,

Nanjing Medical University,

101 Longmian Avenue, Nanjing, Jiangsu 211166, China

Tel: 86-25-86869339

Email: leicao@njmu.edu.cn

**Supplementary figures and legends**


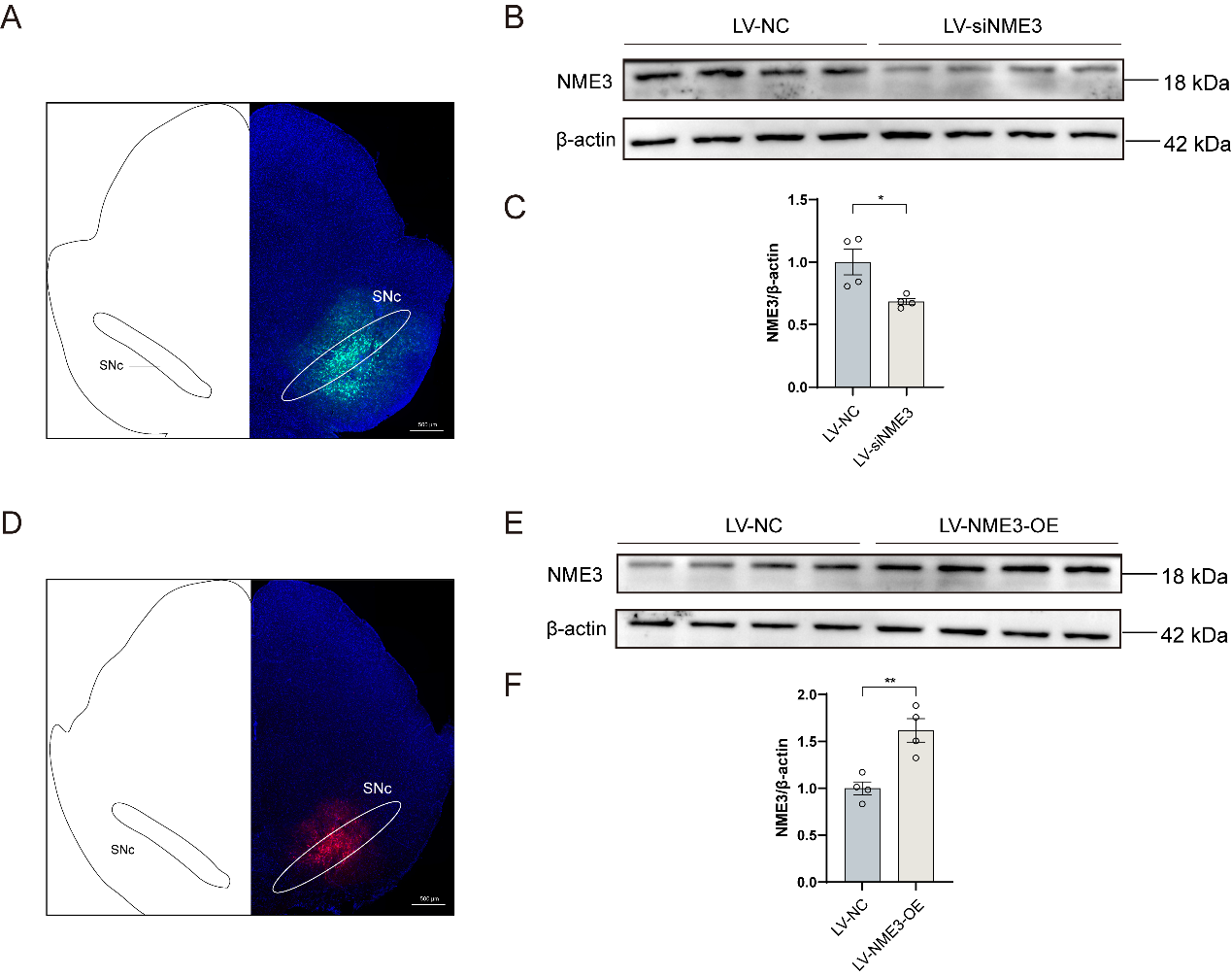


**Figure S1. *In vivo* establishment of NME3 knockdown/overexpression mouse models. A.** Schematic diagram of successful stereotactic intracerebral injection of NME3 knockdown lentivirus. **B**. Representative immunoblot of NME3 protein in the midbrain tissue of mice. **C.** Semi-quantitative analysis of NME3 expression (n = 4 mice per group). **D.** Schematic diagram of stereotactic intracerebral injection of NME3 overexpression lentivirus. **E**. Representative immunoblot of NME3 protein in the midbrain tissue of mice. **F.** Quantitative analysis of NME3 expression (n = 4 mice per group). Data represent mean ± S.E.M. and analyzed by t-test. ^*^*p*<0.05, ^**^*p*<0.01.


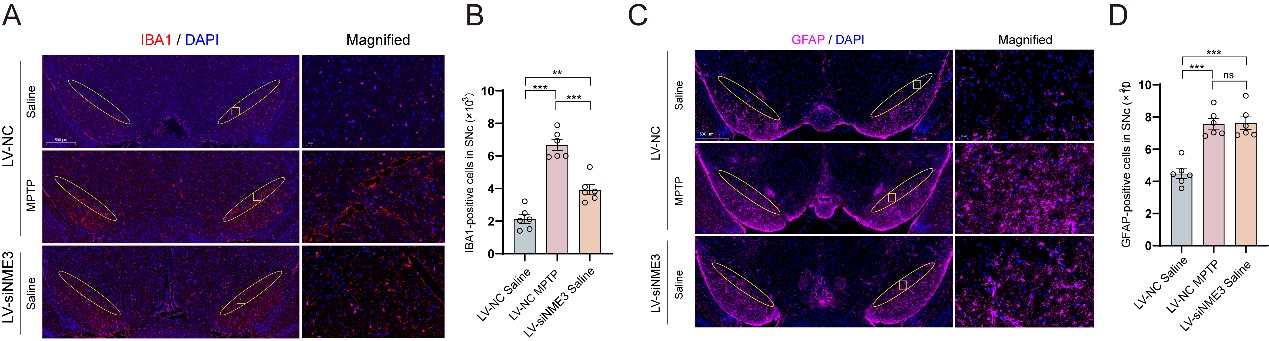


**Figure S2. NME3 knockdown induces abnormal proliferation and activation of glial cells. A.** Representative immunofluorescence images of IBA1 labeling (red) in the SNc region of mice. **B.** Quantitative analysis of IBA1 fluorescence intensity (n = 6 mice per group). **C.** Representative immunofluorescence images of GFAP labeling (purple). **D.** Quantitative analysis of GFAP fluorescence intensity (n = 6 mice per group). Data represent mean ± S.E.M. and analyzed by one-way ANOVA. ^**^*p*<0.01, ^***^*p*<0.001; ns indicates no significant difference.


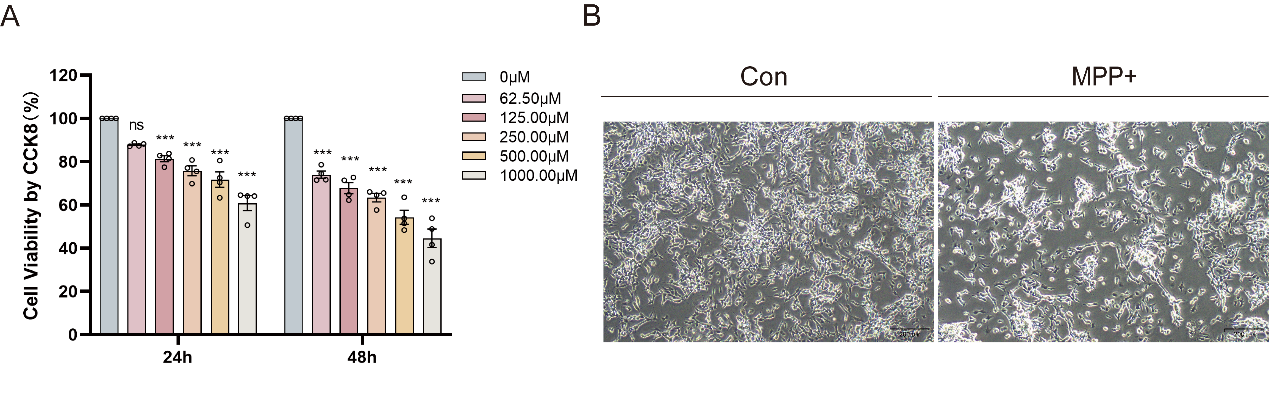


**Figure S3. Establishment of MPP^+^-induced PD cell model and optimization of MPP^+^ concentration and treatment duration. A.** CCK-8 assay assessing SH-SY5Y cell viability under different MPP^+^ concentrations and treatment durations (n = 4 per group). The optimal condition for PD model induction was determined to be 250 μM MPP^+^ for 48 h, which was subsequently used for follow-up cellular experiments. **B.** Representative morphological images of control and MPP^+^-treated model cells. Data represent mean ± S.E.M. and analyzed by one-way ANOVA. ^***^*p*<0.001; ns indicates no significant difference.


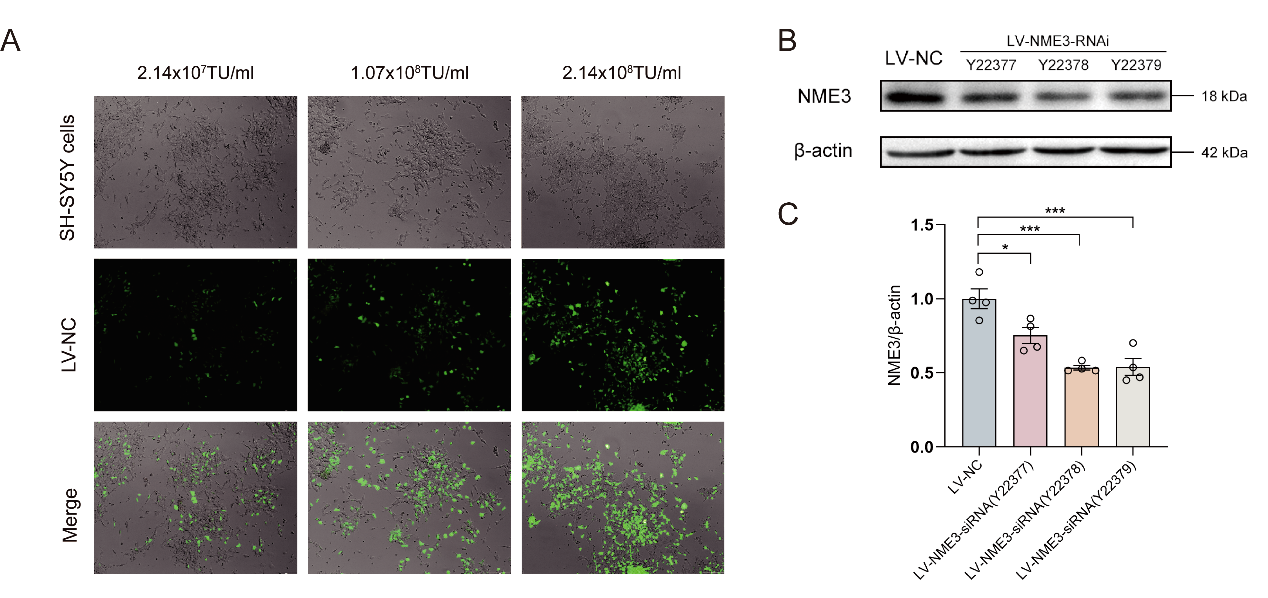


**Figure S4. LV-NME3-siRNA inhibits NME3 expression in SH-SY5Y cells. A.** GFP-positive cells (green) demonstrate lentiviral transfection efficiency, with the optimal titer determined to be 2.14×10⁸ TU/ml for SH-SY5Y cell transduction. **B.** Representative immunoblot of NME3 protein. LV-NME3-siRNA (Y22378) was selected as the optimal lentiviral vector for subsequent experiments. **C.** Semi-quantitative analysis of NME3 expression (n = 4 per group). Data represent mean ± S.E.M. and analyzed by one-way ANOVA. ^*^*p*＜0.05, ^***^*p*<0.001.


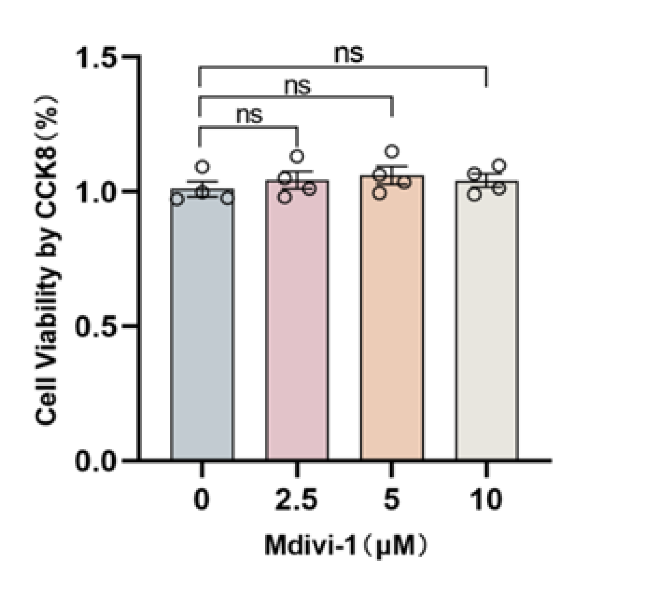


**Figure S5. CCK8 assay evaluates the effects of different Mdivi-1 concentrations on SH-SY5Y cell viability.** Data represent mean ± S.E.M. and analyzed by one-way ANOVA; ns indicates no significant difference.


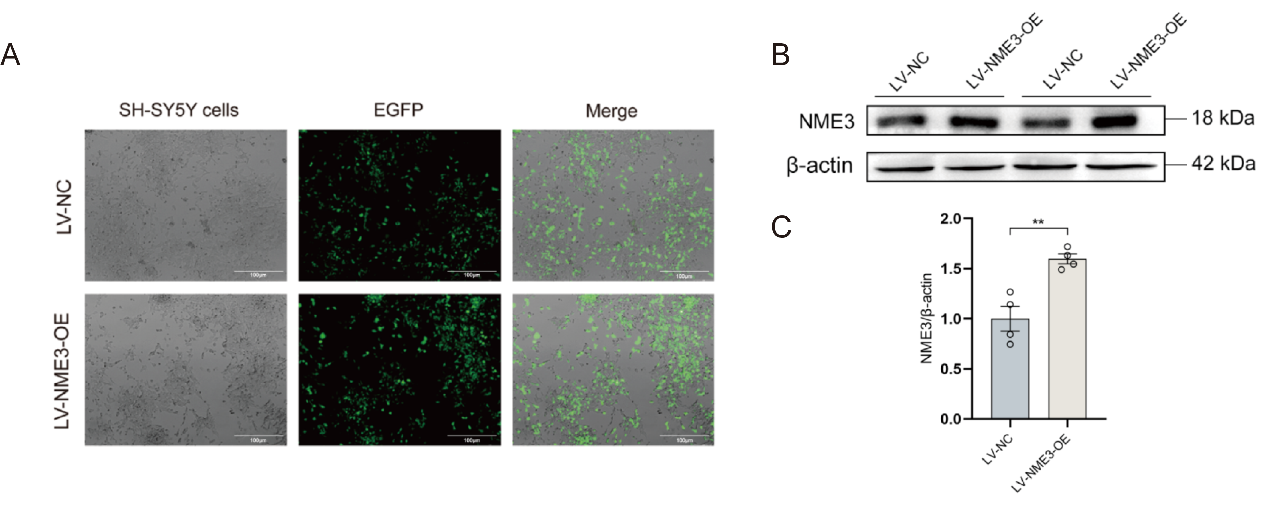


**Figure S6. Lentivirus-packaged LV-NME3-OE upregulates NME3 expression in SH-SY5Y cells. A.** Determination of optimal lentiviral titer for SH-SY5Y cell transduction with GFP (green fluorescent protein) indicating transduction efficiency. **B**. Representative immunoblot of NME3 protein levels. **C**. Quantitative analysis of NME3 expression (n = 4 per group). Data represent mean ± S.E.M. and analyzed by t-test. ^**^*p*<0.01.


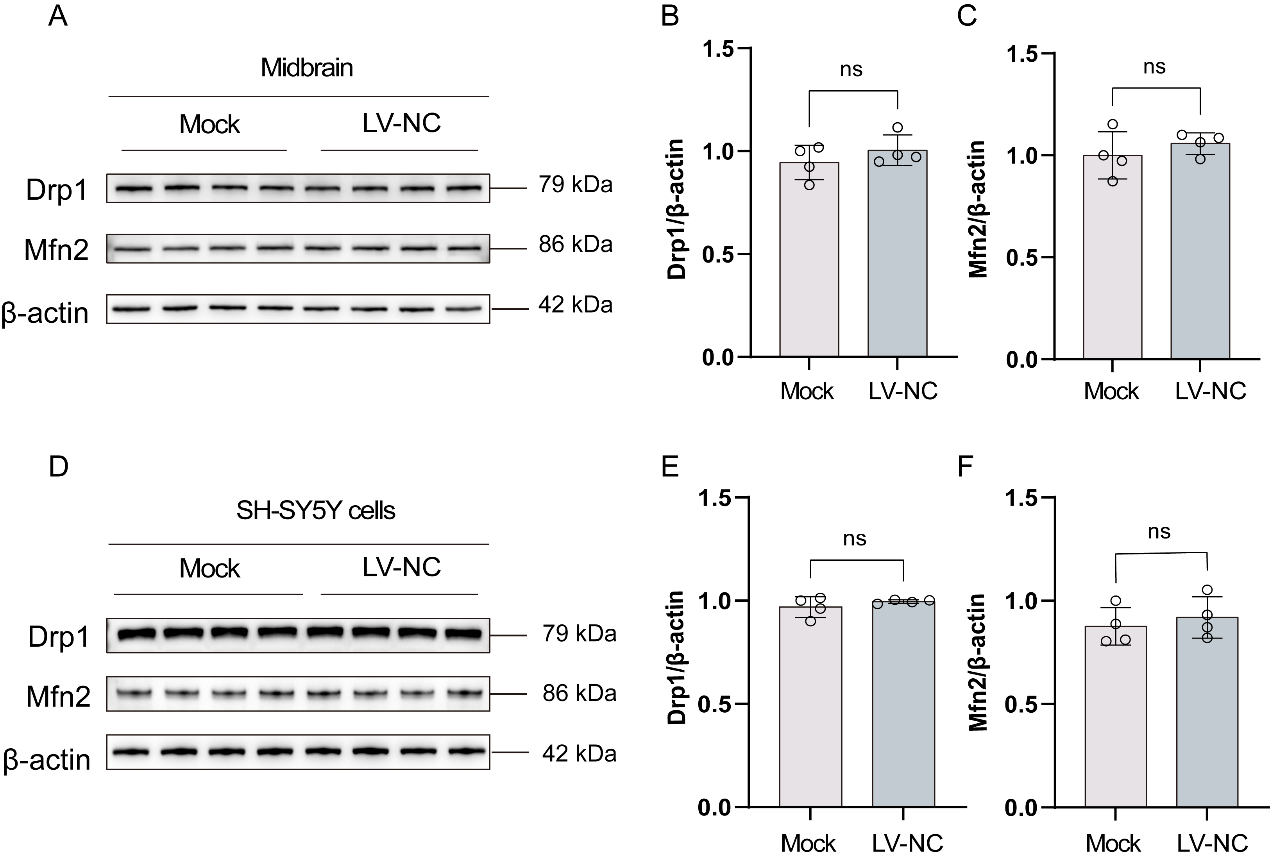


**Figure S7. The untreated control group did not affect the expression of Drp1 or Mfn2 expression *in vivo* and *in vitro*. A.** Western blot analyses of Drp1 and Mfn2 protein expression in midbrain; Mock, the untreated control group; LV-NC, the negative control virus group. **B and C.** Semi-quantitative analysis of Drp1 and Mfn2 expression (n = 4 mice per group). **D**. Western blot analysis of Drp1 and Mfn2 protein expression in SH-SY5Y; Mock, the untreated control group; LV-NC, the negative control virus group. **E and F.** Semi-quantitative analysis of Drp1 and Mfn2 expression (n = 4 mice per group). Data represent mean ± S.E.M. and analyzed by t-test, ns indicates no significant difference.
